# Supplementary material for: Optimal gestational weight gain and pregnancy outcomes, by BMI and height, in a marginalised population of women with short stature living along the Thailand-Myanmar border: A retrospective cohort, 2004–2023
Source: PLoS One. 2025 Oct 3;20(10):e0330256. doi: 10.1371/journal.pone.0330256 (PMC12494276; doi:10.1371/journal.pone.0330256)
Supplement: S1 File — (PDF) [file pone.0330256.s001.pdf]

SUPPLEMENTARY FIGURES AND TABLES

Table S1 Maternal demographic and outcome descriptive statistics by Asia-Pacific BMI categories (overweight and obese groups separated).

**Variable**	**All**	**Underweight (<18.5kg/m²)**	**Normal (18.5 - 22.9kg/m²)**	**Overweight (23 - 24.9kg/m²)**	**Obese (≥ 25kg/m²)**
**Total women**	17194	3019 (17.6)	10115 (58.8)	2085 (12.1)	1975 (11.5)
**Median Age (IQR), years**	25 (20-30)	23 (20-28)	24 (20-30)	27 (22-32)	29 (24-33)
**Nulliparous (%)**	6231 (36.2)	1345 (44.6)	3933 (38.9)	590 (28.3)	363 (18.4)
**Ethnicity**					
Karen (%)	8994 (52.3)	1383 (45.8)	5427 (53.7)	1165 (55.9)	1019 (51.6)
Burmese (%)	4160 (24.2)	884 (29.3)	2154 (21.3)	503 (24.1)	619 (31.3)
Other (%)	4040 (23.5)	752 (24.9)	2534 (25.1)	417 (20.0)	337 (17.1)
**Mean Height (SD), cm**	151.4 (5.4)	152 (5.5)	151.1 (5.4)	151.6 (5.4)	152.1 (5.4)
**Height <153cm (%)**	10205 (59.4)	1692 (56.0)	6198 (61.3)	1205 (57.8)	1110 (56.2)
**Mean GWG (SD), kg**	9.3 (4)	10.2 (3.5)	9.5 (3.8)	8.8 (4.3)	7.5 (4.4)
**NAM GWG Recommendation (kg)**	NA	12.5–18	11.5–16	7–11.5	5–9
Below NAM (%)	10725 (62.4)	2266 (75.1)	7305 (72.2)	666 (31.9)	488 (24.7)
Within NAM (%)	4814 (28.0)	696 (23.1)	2363 (23.4)	902 (43.3)	853 (43.2)
Above NAM (%)	1655 (9.6)	57 (1.9)	447 (4.4)	517 (24.8)	634 (32.1)
**Smokers (%)**	2650 (15.4)	558 (18.5)	1673 (16.5)	258 (12.4)	160 (8.2)
**Hypertensive Disorder of Pregnancy (%)**	1127 (6.6)	110 (3.6)	557 (5.5)	164 (7.9)	296 (15.0)
**Gestational Diabetes Mellitus (%)**	628 (3.7)	77 (2.6)	219 (2.2)	106 (5.1)	226 (11.4)
**Malaria in Pregnancy (%)**	1703 (9.9)	385 (12.8)	1077 (10.6)	150 (7.2)	91 (4.6)
**Caesarean Section (%)**	857 (5.0)	86 (2.8)	405 (4.0)	151 (7.2)	215 (10.9)

Table S2 Neonatal descriptive statistics by maternal Asia-Pacific BMI categories (overweight and obese groups separated).

**Variable**	**All**	**Underweight (<18.5kg/m²)**	**Normal (18.5 - 22.9kg/m²)**	**Overweight (23 - 24.9kg/m²)**	**Obese (≥ 25kg/m²)**
**All births with known outcome (%)**	17194	3019 (17.6)	10115 (58.8)	2085 (12.1)	1975 (11.5)
Live births	17109 (99.5)	2999 (99.3)	10065 (99.5)	2079 (99.7)	1966 (99.5)
Stillbirths	85 (0.5)	20 (0.7)	50 (0.5)	6 (0.3)	9 (0.5)

Male	8648 (50.3)	1494 (49.5)	5096 (50.4)	1061 (50.9)	997 (50.5)
Median EGA (IQR), weeks	39.3 (38.4–40.1)	39.2 (38.3–40)	39.3 (38.4–40.1)	39.4 (38.5–40.2)	39.4 (38.6–40.2)
Preterm births <37 weeks (%)	1048 (6.1)	259 (8.6)	645 (6.4)	90 (4.3)	54 (2.7)
**Birthweight measured <72 hours (%)**	15783	2672 (16.9)	9284 (58.8)	1959 (12.4)	1868 (11.8)
Mean Birthweight (SD), g	2982 (446.8)	2843.5 (440.9)	2959 (434.4)	3085.7 (426.4)	3185.8 (444.8)
SGA (%)	2463 (15.6)	470 (17.6)	1414 (15.2)	288 (14.7)	291 (15.6)
AGA (%)	12402 (78.6)	2061 (77.1)	7333 (79.0)	1561 (79.7)	1447 (77.5)
LGA (%)	918 (5.8)	141 (5.3)	537 (5.8)	110 (5.6)	130 (7.0)

Fig S1. Maternal BMI distribution in the cohort. Bars are coloured according to Asia-Pacific BMI categories; black vertical lines indicate WHO classification cut-offs (at 18.5, 25 and 30kg/m^2).

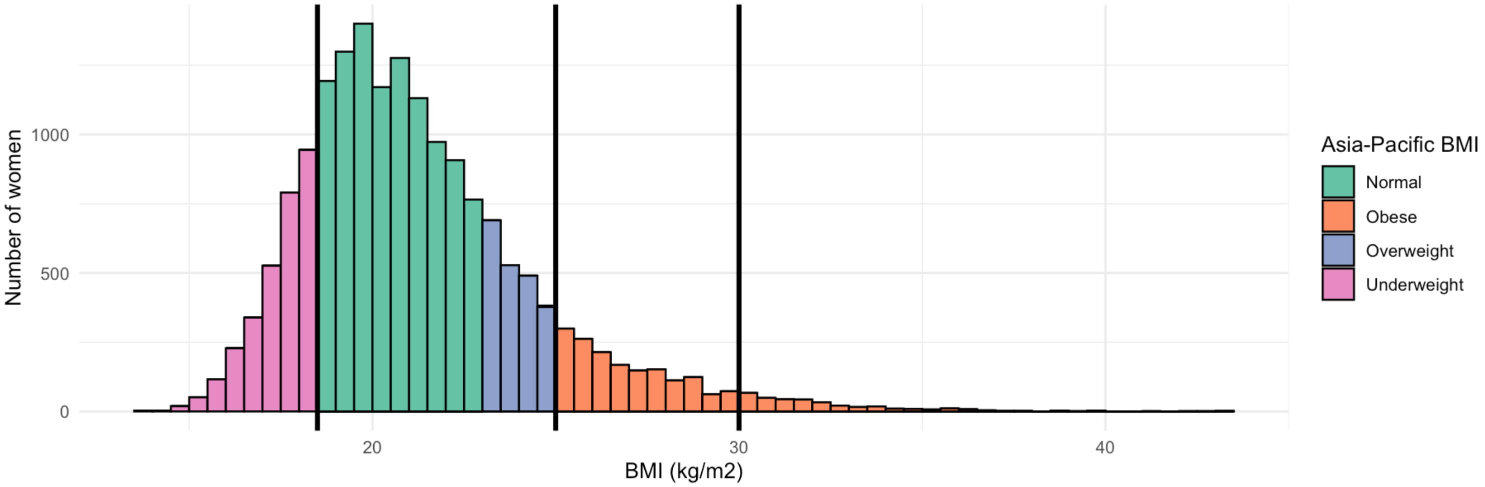

Fig S2. Distribution of height (cm) in the cohort, with red line at height of 153cm, corresponding to the IG-21 study's inclusion cut-off.

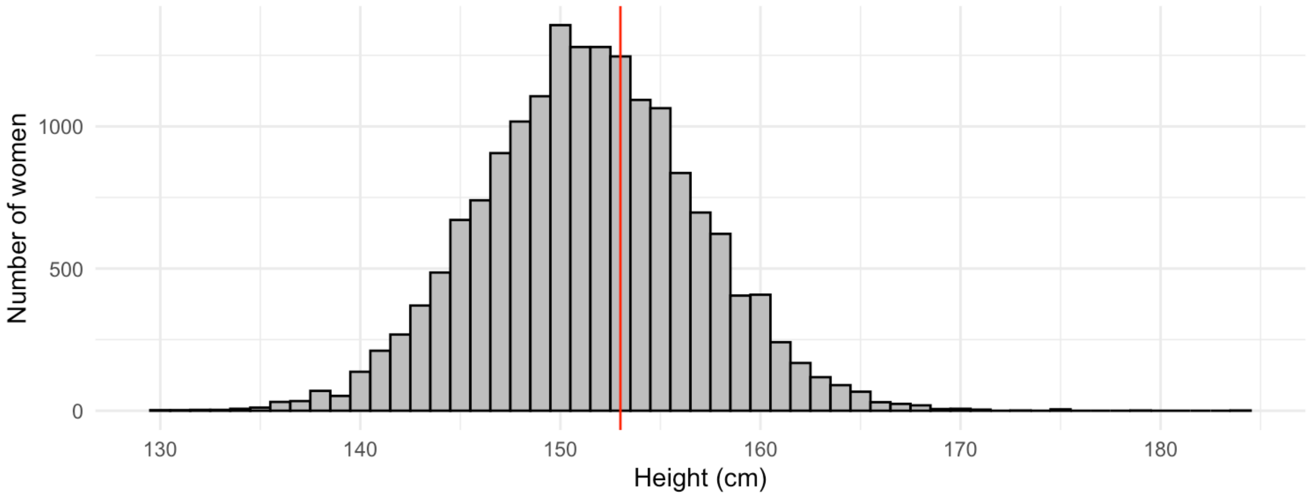

Table S3. Multivariable logistic regression showing adjusted odds ratios (OR, 95% CI, p-value) for maternal and neonatal health outcomes against continuous predictor variables of GWG, BMI, and height. All models adjusted for age & parity combined, smoking status, malaria, ethnicity and year group.

Predictor	HDoP<br>N=1127	GDM<br>N=628	Caesarean Section<br>N=857	Preterm delivery<br>N=1048	SGA<br>N=2463	LGA<br>N=918
GWG<br>(per 10kg change)	1.79<br>(1.54–2.09)<br>**p<0.001**	0.67<br>(0.53–0.83)<br>**p<0.001**	1.64<br>(1.38–1.95)<br>**p<0.001**	0.22<br>(0.19–0.26)<br>**p<0.001**	0.12<br>(0.05–0.24)<br>**p<0.001**	2.25<br>(1.95–2.60)<br>**p<0.001**
BMI (kg/m²)	1.13<br>(1.12–1.15)<br>**p<0.001**	1.12<br>(1.09–1.14)<br>**p<0.001**	1.13<br>(1.10–1.15)<br>**p<0.001**	0.93<br>(0.91–0.95)<br>**p<0.001**	0.93<br>(0.90–0.95)<br>**p<0.001**	1.02<br>(1.00–1.04)<br>p=0.063
Height<br>(per 10cm change)	1.10<br>(0.98–1.23)<br>p=0.098	1.04<br>(0.88–1.21)<br>p=0.660	0.59<br>(0.52–0.67)<br>**p<0.001**	0.85<br>(0.75–0.96)<br>**p=0.007**	0.99<br>(0.92–1.08)<br>p=0.872	0.99<br>(0.89–1.10)<br>p=0.803

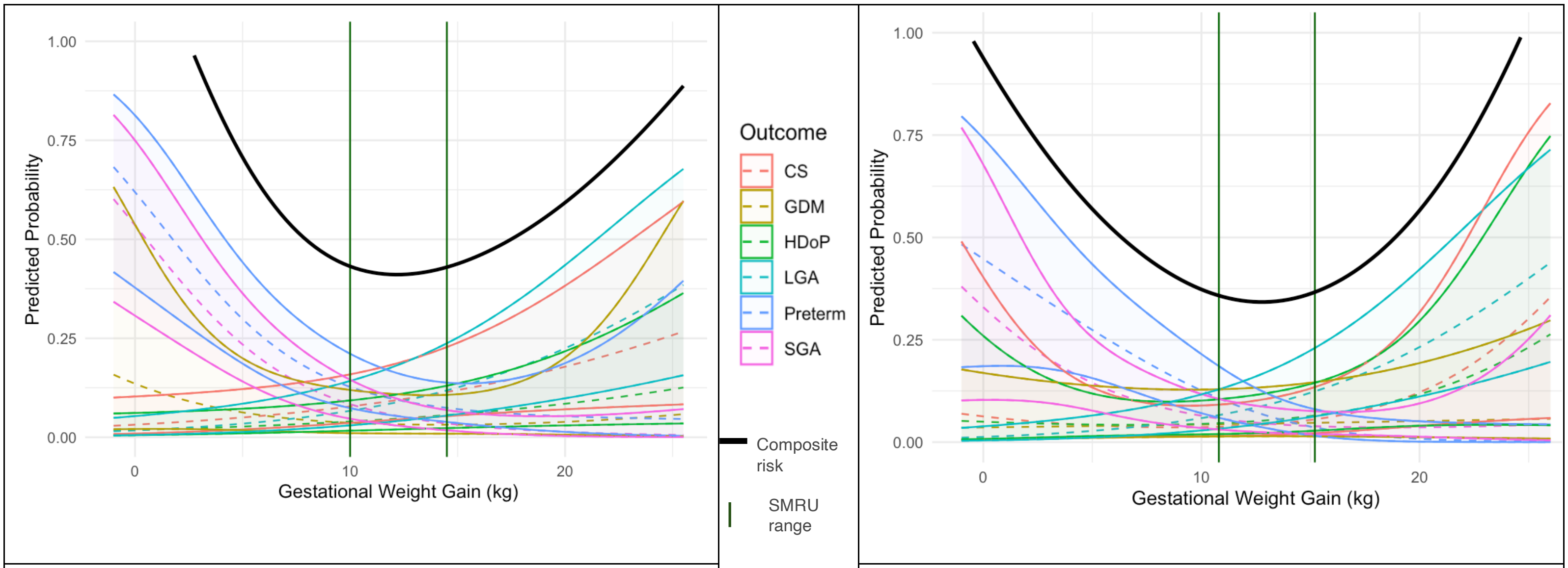

(a) Underweight BMI, height <153cm

(**b**) Underweight BMI, height ≥153cm

Figure S3. Predicted probabilities with 95% confidence intervals (y) of adverse outcomes across GWG (x) for women with a normal first trimester BMI. Calculated using logistic regression models incorporating cubic splines. (**a**) Women with underweight BMI and height <153cm, N=1,692. SMRU GWG recommendations superimposed as vertical green lines at 10.0 and 14.5kg; (**b**) Women with underweight BMI and height ≥153cm, N=1,327. SMRU GWG recommendations superimposed as vertical green lines at 11.0 and 15.1kg.

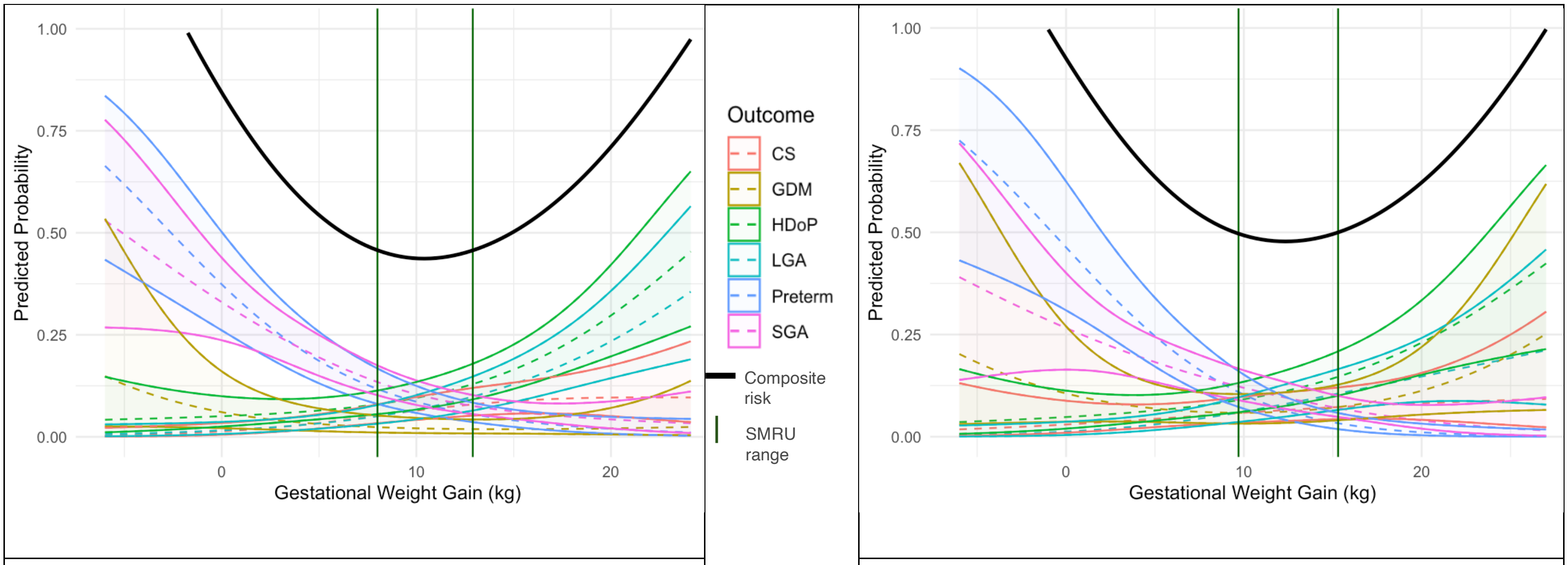

(a) Normal BMI, height <153cm

(**b**) Normal BMI, height ≥153cm

Figure S4. Predicted probabilities with 95% confidence intervals (y) of adverse outcomes across GWG (x) for women with a normal first trimester BMI. Calculated using logistic regression models incorporating cubic splines. (**a**) Women with normal BMI and height <153cm, N=6,198. SMRU GWG recommendations superimposed as vertical green lines at 8.0 and 12.9kg (**b**) Women with normal BMI and height ≥153cm, N=3,917. Calculated SMRU GWG recommendations superimposed as vertical green lines at 9.7 and 15.3kg.

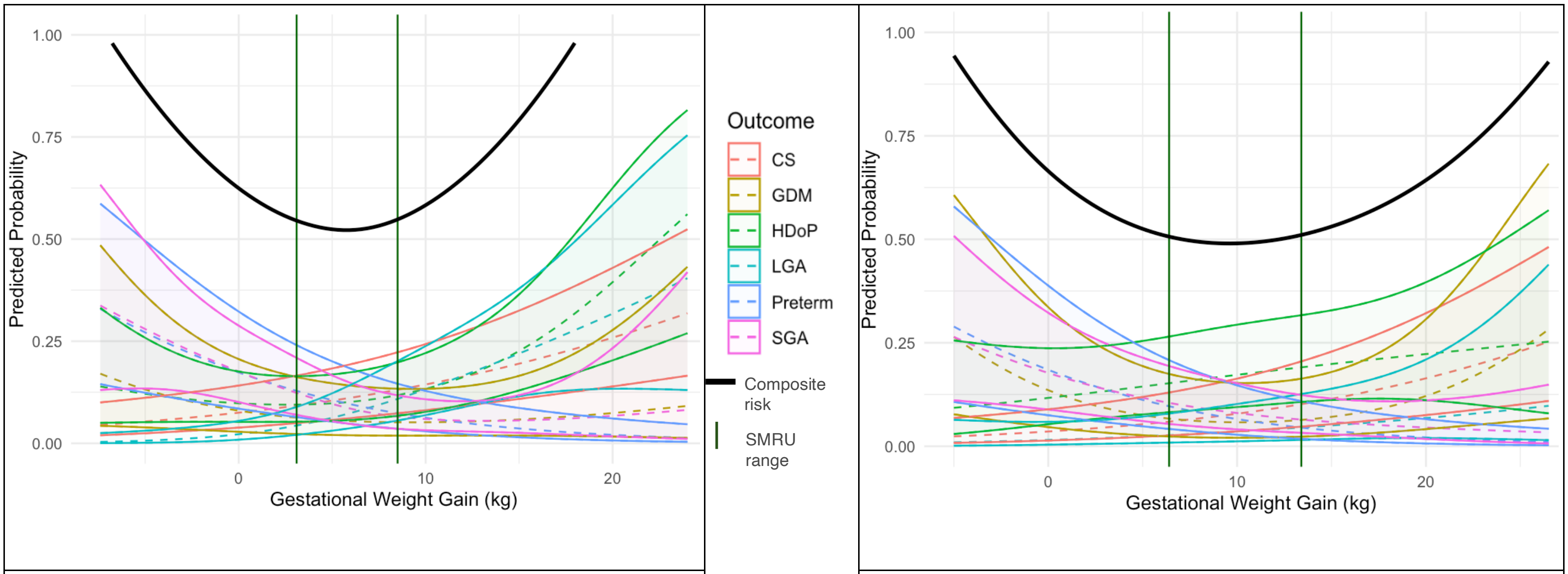

(a) Overweight/obese BMI, height <153cm

(**b**) Overweight/obese BMI, height ≥153cm

Figure S5. Predicted probabilities with 95% confidence intervals (y) of adverse outcomes across GWG (x) for women with an overweight/obese first trimester BMI. Calculated using logistic regression models incorporating cubic splines. (**a**) Women with overweight or obese BMI and height <153cm, N=2,315. SMRU GWG recommendations superimposed as vertical green lines at 3.1 and 8.5kg (**b**) Women with overweight or obese BMI and height ≥153cm, N=1,745. SMRU GWG recommendations superimposed as vertical green lines at 6.4 and 13.4kg.
