# Supplementary material for: Optimal gestational weight gain and pregnancy outcomes, by BMI and height, in a marginalised population of women with short stature living along the Thailand-Myanmar border: A retrospective cohort, 2004–2023
Source: PLoS One. 2025 Oct 3;20(10):e0330256. doi: 10.1371/journal.pone.0330256 (PMC12494276; doi:10.1371/journal.pone.0330256)
Supplement: Attachment — Submitted filename: PONE-D-24-59010_R1_Reviewer 2.pdf [file pone.0330256.s003.pdf]

PLOS ONE

Optimal gestational weight gain and pregnancy outcomes, by BMI and height, in a marginalised population of women with short stature living along the Thailand-Myanmar border: a retrospective cohort, 2004-2023.

--Manuscript Draft--

Manuscript Number:	PONE-D-24-59010R1
Article Type:	Research Article
Full Title:	Optimal gestational weight gain and pregnancy outcomes, by BMI and height, in a marginalised population of women with short stature living along the Thailand-Myanmar border: a retrospective cohort, 2004-2023.
Short Title:	Gestational Weight Gain by BMI and height in a marginalized population along the Thailand-Myanmar border.
Corresponding Author:	Rose McGready<br>Shoklo Malaria Research Unit<br>Mae Sot, Tak THAILAND
Keywords:	Gestational weight gain; Asia-pacific body mass index; short-stature; migrant; refugee; Small for gestational age; perinatal nutrition; Maternal outcomes
Abstract:	Background<br>Existing gestational weight gain (GWG) standards may not be applicable to women of short stature and those who have limited access to healthcare and are vulnerable to compromised nutrition during pregnancy. To inform the development of population-specific recommendations, this study investigated optimal GWG, by height and Body Mass Index (BMI), in a minority migrant and refugee population living along the Thailand-Myanmar border.<br><br>Methods<br>Records of all women attending antenatal care in the first trimester at the Shoklo-Malaria Research Unit between 2004 and 2023 were retrospectively examined. GWG of 17,194 women was assessed against maternal, delivery and neonatal outcomes, by height and per Asia-Pacific BMI category. The Gestation Related Optimal Weight centiles were used to classify small, appropriate and large for gestational age neonates. Multivariable logistic regression analysis, including natural cubic splines, was used to assess the relationships between GWG and outcomes of interest. Optimal GWG per BMI group was defined as the GWG associated with the lowest composite risk for adverse outcomes. The optimal range included GWG values that did not exceed a 5% increase from the corresponding minimum composite risk.<br><br>Results<br>Optimal GWG in women shorter than 153cm was lower per BMI group than the National Academy of Medicine and Intergrowth-21 recommendations: underweight 12.1kg (10.0-14.5), normal 10.4kg (8.0-12.9), overweight/obese 5.3kg (3.1-8.5); but comparable for women 153cm or more: underweight: 13.1kg (11.0–15.1), normal: 12.3kg (9.7-15.3), overweight/obese: 9.5kg (6.4-13.4).<br><br>Conclusion<br>Optimal GWG ranges are lower for this population with short stature compared to existing international guidelines. Clinical and contextual factors must be considered when implementing GWG recommendations for this, and other marginalized and short-stature populations.
Order of Authors:	Mary Gouws
	Rose McGready
	Wirichada Pan-ngum
	Aung Myat Min
	Nay Win Tun

Powered by Editorial Manager® and ProduXion Manager® from Aries Systems Corporation

	Mary Ellen Gilder
	Taco Jan Prins
	Widi Yotyingaphiram
	Mupawjay Pimanpanarak
	Jacher Viladpai-Nguen
	Nuttapol Panachuenwongsakul
	François H Nosten
	Sue J Lee
**Response to Reviewers:**	Dear Dr Tennekoon<br><br>Re: Response to reviewer comments PLOS ONE-D-24-59010<br>Optimal gestational weight gain and pregnancy outcomes, by BMI and height, in a marginalised population of women with short stature living along the Thailand-Myanmar border: a retrospective cohort, 2004-2023.<br><br>On behalf of the authors of the above manuscript, I would like to submit a revised version of this manuscript. We thank the reviewers for their constructive comments which we feel has strengthened our manuscript. We have addressed all comments as specified in the bullet points overleaf. We have uploaded 2 new versions of the manuscript: one with tracked changes and one unmarked, and labelled them as prescribed by PLOS ONE.<br><br>The requested amended funding statement is as follows:<br>The Shoklo Malaria Research Unit is supported by the Wellcome-Trust Major Overseas Programme in Southeast Asia (#220211, lead applicant Nicholas Day) and in the years prior to that (WT-106698). MG was supported by a Rhodes Scholarship. For the purposes of Open Access, the author has applied a CC BY public copyright license to any Author Accepted Manuscript version arising from this submission. The funders had no role in study design, data collection and analysis, decision to publish, or preparation of the manuscript. There was no additional external funding received for this study.<br><br>We hope very much that this revised and improved manuscript will be acceptable for publication.<br><br>Yours sincerely,<br><br>Mary Gouws<br>DPhil Candidate, University of Oxford.<br>MSc International Health and Tropical Medicine, University of Oxford.<br>MBChB, University of Cape Town.<br><br>EDITORS' SPECIFIC POINTS:<br>Please find responses to comments in blue.<br><br><br>1.Please ensure that your manuscript meets PLOS ONE's style requirements, including those for file naming. The PLOS ONE style templates can be found at<br><br>The manuscript has been updated to meet PLOS ONE’s style requirements and figures and supporting information have been saved with appropriate names and are now uploaded separately as individual files.<br><br>2.Thank you for stating in your Funding Statement: “The Shoklo Malaria Research Unit is supported in part by the Wellcome-Trust Major Overseas Programme in Southeast Asia (# 220211, https://doi.org/10.35802/220211; lead applicant Nicholas Day). For the purpose of Open Access, the author has applied a CC BY public copyright licence to any Author Accepted Manuscript version arising from this submission. There was no additional external funding received for this study. The funders had no role in study design, data collection and analysis, decision to publish, or preparation of the

manuscript."
Please provide an amended statement that declares *all* the funding or sources of support (whether external or internal to your organization) received during this study, as detailed online in our guide for authors at http://journals.plos.org/plosone/s/submit-now. Please also include the statement "There was no additional external funding received for this study." in your updated Funding Statement.
Please include your amended Funding Statement within your cover letter. We will change the online submission form on your behalf.
We have amended the funding statement (below) and have included this revised version in the cover letter.
"The Shoklo Malaria Research Unit is supported by the Wellcome-Trust Major Overseas Programme in Southeast Asia (#220211, lead applicant Nicholas Day) and in the years prior to that (WT-106698). MG was supported by a Rhodes Scholarship. For the purposes of Open Access, the author has applied a CC BY public copyright license to any Author Accepted Manuscript version arising from this submission. The funders had no role in study design, data collection and analysis, decision to publish, or preparation of the manuscript. There was no additional external funding received for this study."

3.We note that you have indicated that there are restrictions to data sharing for this study. For studies involving human research participant data or other sensitive data, we encourage authors to share de-identified or anonymized data. However, when data cannot be publicly shared for ethical reasons, we allow authors to make their data sets available upon request. For information on unacceptable data access restrictions, please see http://journals.plos.org/plosone/s/data-availability#loc-unacceptable-data-access-restrictions.
Before we proceed with your manuscript, please address the following prompts:
a)If there are ethical or legal restrictions on sharing a de-identified data set, please explain them in detail (e.g., data contain potentially identifying or sensitive patient information, data are owned by a third-party organization, etc.) and who has imposed them (e.g., a Research Ethics Committee or Institutional Review Board, etc.). Please also provide contact information for a data access committee, ethics committee, or other institutional body to which data requests may be sent.
b)If there are no restrictions, please upload the minimal anonymized data set necessary to replicate your study findings to a stable, public repository and provide us with the relevant URLs, DOIs, or accession numbers. Please see http://www.bmj.com/content/340/bmj.c181.long for guidelines on how to de-identify and prepare clinical data for publication. For a list of recommended repositories, please see https://journals.plos.org/plosone/s/recommended-repositories. You also have the option of uploading the data as Supporting Information files, but we would recommend depositing data directly to a data repository if possible.

Please update your Data Availability statement in the submission form accordingly.
The data availability statement now reads: "The data cannot be shared publicly due to ethical restrictions: this data was routinely collected from a marginalized population of undocumented refugees and migrants and the women have not consented for the data to be shared. These restrictions are in keeping with the policy of the Oxford Tropical Research Ethics Committee. However, de-identified data is available from the Mahidol-Oxford Research Unit institutional data access committee upon reasonable request from researchers who meet the criteria for access to confidential data (contact Rita Chanviriyavuth, email rita@tropmedres.ac)."

4.For studies involving third-party data, we encourage authors to share any data specific to their analyses that they can legally distribute. PLOS recognizes, however, that authors may be using third-party data they do not have the rights to share. When third-party data cannot be publicly shared, authors must provide all information necessary for interested researchers to apply to gain access to the data. (https://journals.plos.org/plosone/s/data-availability#loc-acceptable-data-access-restrictions)
For any third-party data that the authors cannot legally distribute, they should include the following information in their Data Availability Statement upon submission:
1) A description of the data set and the third-party source
2) If applicable, verification of permission to use the data set
3) Confirmation of whether the authors received any special privileges in accessing the

data that other researchers would not have
4) All necessary contact information others would need to apply to gain access to the data
This is not applicable for our study: third-party data was not involved in this analysis.

5.PLOS requires an ORCID iD for the corresponding author in Editorial Manager on papers submitted after December 6th, 2016. Please ensure that you have an ORCID iD and that it is validated in Editorial Manager. To do this, go to 'Update my Information' (in the upper left-hand corner of the main menu), and click on the Fetch/Validate link next to the ORCID field. This will take you to the ORCID site and allow you to create a new iD or authenticate a pre-existing iD in Editorial Manager.
Rose McGready is the corresponding author, with ORCID iD: 0000-0003-1621-3257.

6. Please amend the manuscript submission data (via Edit Submission) to include author "Jacher Viladpai-Nguen".
This has been updated accordingly, as requested.

RESPONSES TO REVIEWER COMMENTS
Reviewer #1: Thank for the paper, give us a good presentation, have an excellent proposed to help a vulnerable group of pregnant women. Nevertheless, I have a few suggestions before their publication.

Unify the objective (line 30-31) from the abstract with the last section of introduction (lines 71-72)
Amended as suggested.
Line 140. Is SES socioeconomic status? Clarify
SES is socioeconomic status and this is now spelled out in the methods.
Add the information to literacy and socioeconomic status as limitation (in Discussion section), because these variables can have influence on the health and well-being of the pregnant women and affect their GWG.
Amended as suggested (lines 285-288).

What is the reason to have greater intervale in those women with overweight-and obesity pregestational BMI (Table 3)? Add the information in discussion section.
This wider interval can be attributed to the wide variation of first trimester BMI in this overweight and obese group, ranging from 23 to 43kg/m² (a total range of 20 kg/m²). In the underweight BMI group, the range was only 4.9 kg/m² (13.5 to 18.4 kg/m²) and the range for the normal BMI category was bound at 4.4 kg/m² (18.5-22.9 kg/m²). We have added a few lines to discuss this, as suggested (lines 277-280) of the manuscript.

My final doubt is the applicability can be effective in all women with short stature? Regardless of the access to health service, and another sociocultural context? Or only those from Nepal, East Timor and Bangladesh?
There are limited data globally on women with short stature and as far as we are aware, no guidelines for GWG currently exist for these groups. Thus, the findings from our study currently provide the only insight for any pregnant woman with short stature, regardless of access to health services and sociocultural contexts. This has been clarified in the manuscript (lines 73-74 and 328-329).

Reviewer #2: Dear Authors,
Thank you for the opportunity to review this interesting piece of work, that highlights an under-adressed data gap in a marginalized population.

However, I have a few minor concerns and queries about the article:

1) Firstly, I suggest adjustments to the wordings in the article, particularly on the usage of "short stature women", as it may come across as stigmatizing this population, taking into consideration this population may have shorter stature due to a underlying varying genetic and/or environmental reasons. I suggest adhering to person-first language i.e. "women with shorter stature" or "women with short stature" for a more neutral tone.
Thank you for this suggestion. Have amended the manuscript throughout to adhere to first person language.

2) I am curious as to why neonates with major congenital abnormalities were excluded

from the analysis, considering maternal obesity is a known risk factor for congenital anomalies. If there is a strong reason to this, I suggest the authors include the explanation in the main text.
Thank you for raising this. The primary reason for this exclusion is that neonates with major congenital abnormalities often present with skewed birth weight distributions, such as being small or large for gestational age, which could confound the assessment of the relationship between maternal weight gain and neonatal outcomes. Additionally, certain congenital abnormalities can directly or indirectly influence gestational weight gain (GWG), further complicating the interpretation of results if included. As there was a relatively small number of neonates with congenital abnormalities, we anticipate that excluding this group should not substantially affect the results in any case. As suggested, we have added an explanation of this to the methods section (lines 109-112).

3) The women in the overweight/obese BMI range were analyzed as a homogenous group rather than 2 separate groups. The authors have alluded to the inherent issue of analyzing BMI as a categorical factor rather than continuous - that it oversimplifies risk estimation for women within a broad range of profiles such as BMI. In fact, the paper by Morisaki, quoted by the authors, found rather differing optimal GWG for women in the overweight (7.7kg) vs obese (4.3 kg) category. Therefore, I suggest the authors consider splitting the analysis of overweight (BMI 23-24.9 kg/m2) and obese (>25 kg/m2) categories, for better clinical applicability.
This is an important issue and we thank the reviewer for raising it. Although growing, the overweight and obese groups are still relatively small populations in this area. The small numbers meant that when separated, the samples were underpowered and we were not able to produce meaningful results. To demonstrate this more clearly, we have added Table 1 and 2 repeated but separated into overweight and obese groups in the supplementary materials (Table S1 and Table S2). The number of overweight BMI women was 2085 and 1975 for obese women. Additionally, the Asia-pacific classification of overweight BMI (BMI 23-24.9 kg/m2) is very narrow. We have mentioned this as a limitation in the discussion section.

4) I suggest p-values to be included in Tables 1 and 2, to reflect the baseline differences among different BMI categories, if any, as they may hold weight in interpreting the main outcome of the analysis.
Thank you for this suggestion. After consulting with the study statistician and in accordance with the STROBE guidelines for observational studies (section 14, 2007 Explanation and Elaboration by Vandenbroucke et al.) we note that descriptive tables are recommended to summarize participant characteristics without inferential statistics such as p-values. The rationale is to prevent any implication that these tables test hypotheses or draw conclusions about the data beyond simple description.

5) The authors addressed a potential overestimation of risk of adverse outcomes using the composite score - my question is how should the reader account for this when applying the findings of the analysis in clinical practice. Should the clinician advise for a more lenient range, or perhaps upper-half of the normal range reported in this study, while advising optimal GWG for women with shorter stature? I suggest the authors to discuss the clinical implications of this.
We would suggest that a clinician in this setting should advise the same GWG range for all women within each BMI group, based on height. Because two thirds of our pregnancies had no adverse outcomes (n=11,309, 65.8%), these would have had the largest influence on the GWG estimations. Less than 6% had more than 1 of the adverse outcomes indicating not only that it was uncommon for women to experience more than 1 adverse outcome, but also that those who did would have minimal impact on the estimated GWG recommendation. The frequencies of pregnancies experiencing no adverse outcome, 1 adverse outcome and 1 or more adverse outcomes is also now included in the results and more explanation has been included in the discussion as well.

Reviewer #3: The study explored optimal gestational weight gain (GWG) by BMI and height in a marginalized migrant population along the Thailand-Myanmar border, aiming to inform more population-specific guidelines. Here are some of my concerns that require addressing:

The study does not distinguish between iatrogenic (medically indicated) and spontaneous preterm birth. Iatrogenic PTB (e.g., for preeclampsia or fetal distress) is often an appropriate and protective medical intervention rather than an adverse outcome. If iatrogenic PTB is grouped with spontaneous PTB, it could misrepresent GWG's true impact on PTB risk or the composite risk outcome.
Thank you for raising this point. In order to avoid the issues you mention, we have removed iatrogenic PTBs from the dataset (N=174) and rerun the analyses (N=17194).

The study states that they adjusted for GDM screening method changes over time, but does not clearly define how GDM itself was accounted for in their models. A sensitivity analysis excluding women with GDM could strengthen the findings. This is hared across the other pregnancy complications including Malaria, and hypertensive disorder.
A sensitivity analysis excluding women with GDM was run and the GWG ranges were 10.2 to 14.8kg (compared with 10.2-14.8kg) for underweight women, 8.3 to 14.3kg (compared with 8.3 to 14.0kg) for normal weight women and 5.6 to 12.8kg (compared with 5.6 to 12.1kg) for overweight and obese women, i.e., there were no substantial changes to the results as currently presented.
In addition, we have now adjusted for year, and how GDM screening method changed over time in all models (explained in Statistical analysis section in Methods).

Parity status - intriguing the majority of women were obese were multiparous. Nulliparity is what essentially creates a "unknown" risk - if you had "normal deliveries/pregnancies" before then that is the best predictor about cows in future pregnancies. I would be interested in a nulliparous analysis being highlighted. Similarly, inter pregnancy weight gain - if that is something that can be teased out and its associated risk with the composite risk - that is very interesting.
We agree that both these points would be interesting topics to pursue. We have run the analysis in nulliparous women (N=6231) only, and while slightly lower for the underweight and normal BMI groups, the results were not substantially different from the results of the total cohort:
•Underweight BMI group: 10.0-14.3kg (N=1345)
•Normal BMI group: 7.7-12.7kg (N=3933)
•Overweight/obese BMI group: 4.9-11.3 (N=953)
As the differences were minimal, and guideline implementation is already challenging in particular in resource-limited settings, at present we believe that it would not be practical to implement different recommendations across multiple groups. If recommendations become too complicated, the trade-off is often that they are simply not implemented. The next plan is to invite the frontline staff in this resource-limited setting to participate to a working group to determine their perceptions of implementing GWG guidelines, including whether it would be clinically feasible to implement appropriate separate recommendations by parity status.
Unfortunately, we do not have data on interpregnancy weight gain but will keep it mind for future research projects!

I assume - although. this is not spelt out by the authors - that some women here would feature multiple times? If you only included one pregnancy which one? Or how were the repeat pregnancies handled—were they accounted for in the statistical models?
Ideally, we would like to adjust for repeat pregnancies (non-independence of data). However, data on repeat pregnancies only started being collected from 2011/2012 (with site by site initiation) which meant that more than 9,000 pregnancies did not have this information. We have added several sentences to the discussion mentioning this as a limitation (lines 289-291).

The authors use the Perinatal Institute's Gestation Related Optimal Weight (GROW) customised bulk centile calculator V.8.0[18]. They state: GROW offers the advantage of region-specific classification and has coefficients to represent over 100 country-of-origin groups. -> which ethnic group did you choose? and do justify the choice.
Apologies that this was not clearly stated. For women with Burmese ethnicity, the Myanmar standards were used. For Karen and other ethnicities, Nepalese standards were used as the average height of Karen and other ethnicities in the dataset most closely matched the average height of Nepalese women. The methods section has been revised to include this information.

	Strengths:<br>Addresses a population often underrepresented in research.<br>Attempts to tailor GWG recommendations to short-stature women, which is clinically relevant.<br>Uses GROW centiles, which allow for ethnicity-specific fetal growth assessment.<br>Utilizes modern statistical methods (natural cubic splines) to model nonlinear relationships.<br>Thank you!<br><br>Key Limitations to Address Before Publication:<br>Clarify whether multiple pregnancies from the same individuals were included and account for within-subject correlation.<br>Addressed, please see above.<br><br>Differentiate between iatrogenic vs. spontaneous PTB.<br>Addressed, please see above.<br><br>Explicitly describe how GDM was adjusted for in the analysis.<br>Addressed, please see above.<br><br>Provide further justification for cubic spline modelling choices and potential overfitting concerns.<br>We have adjusted the text in the methods to read: "As visual inspection of the association between GWG and adverse outcomes indicated nonlinear relationships, natural cubic splines with logistic regression models were used. Natural cubic splines were used to mitigate overfitting, especially at the boundaries of the data." And the following reference that offers support for this approach is cited: Gauthier J, Wu QV, Gooley TA. Cubic splines to model relationships between continuous variables and outcomes: a guide for clinicians. Bone Marrow Transplant. 2020;55: 675–680. doi:10.1038/s41409-019-0679-x
**Additional Information:**	
**Question**	**Response**
**Financial Disclosure**<br><br>Enter a financial disclosure statement that describes the sources of funding for the work included in this submission. Review the submission guidelines for detailed requirements. View published research articles from *PLOS ONE* for specific examples.<br><br>This statement is required for submission and **will appear in the published article** if the submission is accepted. Please make sure it is accurate.	Yes

**Funded studies**<br>Enter a statement with the following details:<br>• Initials of the authors who received each award<br>• Grant numbers awarded to each author<br>• The full name of each funder<br>• URL of each funder website<br>• Did the sponsors or funders play any role in the study design, data collection and analysis, decision to publish, or preparation of the manuscript?<br><br>Did you receive funding for this work?	
Please add funding details.<br>as follow-up to "**Financial Disclosure**<br><br>Enter a financial disclosure statement that describes the sources of funding for the work included in this submission. Review the submission guidelines for detailed requirements. View published research articles from *PLOS ONE* for specific examples.<br><br>This statement is required for submission and **will appear in the published article** if the submission is accepted. Please make sure it is accurate.<br><br>**Funded studies**<br>Enter a statement with the following details:<br>• Initials of the authors who received each award<br>• Grant numbers awarded to each author<br>• The full name of each funder<br>• URL of each funder website<br>• Did the sponsors or funders play any role in the study design, data collection and analysis, decision to publish, or preparation of the manuscript?<br><br>Did you receive funding for this work?"	The Shoklo Malaria Research Unit is supported in part by the Wellcome-Trust Major Overseas Programme in Southeast Asia (# 220211, https://doi.org/10.35802/220211; lead applicant Nicholas Day). For the purpose of Open Access, the author has applied a CC BY public copyright licence to any Author Accepted Manuscript version arising from this submission. There was no additional external funding received for this study. The funders had no role in study design, data collection and analysis, decision to publish, or preparation of the manuscript.
Please select the country of your main research funder (please select carefully as in some cases this is used in fee calculation).	UNITED KINGDOM - GB

as follow-up to "**Financial Disclosure**<br><br>Enter a financial disclosure statement that describes the sources of funding for the work included in this submission. Review the submission guidelines for detailed requirements. View published research articles from *PLOS ONE* for specific examples.<br><br>This statement is required for submission and **will appear in the published article** if the submission is accepted. Please make sure it is accurate.<br><br>**Funded studies**<br>Enter a statement with the following details:<br>• Initials of the authors who received each award<br>• Grant numbers awarded to each author<br>• The full name of each funder<br>• URL of each funder website<br>• Did the sponsors or funders play any role in the study design, data collection and analysis, decision to publish, or preparation of the manuscript?<br><br>Did you receive funding for this work?"	
**Competing Interests**<br><br>Use the instructions below to enter a competing interest statement for this submission. On behalf of all authors, disclose any competing interests that could be perceived to bias this work—acknowledging all financial support and any other relevant financial or non-financial competing interests.<br><br>This statement is required for submission and **will appear in the published article** if the submission is accepted. Please make sure it is accurate and that any funding sources listed in your Funding Information later in the submission form are also declared in your Financial Disclosure statement.	The authors have declared that no competing interests exist.

View published research articles from *PLOS ONE* for specific examples.<br><br>**NO authors have competing interests**<br><br>Enter: *The authors have declared that no competing interests exist.*<br><br>**Authors with competing interests**<br><br>Enter competing interest details beginning with this statement:<br><br>*I have read the journal's policy and the authors of this manuscript have the following competing interests: [insert competing interests here]*<br><br>* typeset	
**Ethics Statement**<br><br>Enter an ethics statement for this submission. This statement is required if the study involved:<br><br>• Human participants<br>• Human specimens or tissue<br>• Vertebrate animals or cephalopods<br>• Vertebrate embryos or tissues<br>• Field research<br><br>Write "N/A" if the submission does not require an ethics statement.<br><br>General guidance is provided below. Consult the submission guidelines for detailed instructions. **Make sure that all information entered here is included in the Methods section of the manuscript.**	Ethical approval was obtained through Oxford Tropical Research Ethics Committee (OxTREC reference 531-24) and the local body representing the community: Tak Province Community Advisory Board; T-CAB (TCAB202405).

**Format for specific study types**<br><br>**Human Subject Research (involving human participants and/or tissue)**<br>• Give the name of the institutional review board or ethics committee that approved the study<br>• Include the approval number and/or a statement indicating approval of this research<br>• Indicate the form of consent obtained (written/oral) or the reason that consent was not obtained (e.g. the data were analyzed anonymously)<br><br>**Animal Research (involving vertebrate animals, embryos or tissues)**<br>• Provide the name of the Institutional Animal Care and Use Committee (IACUC) or other relevant ethics board that reviewed the study protocol, and indicate whether they approved this research or granted a formal waiver of ethical approval<br>• Include an approval number if one was obtained<br>• If the study involved *non-human primates*, add *additional details* about animal welfare and steps taken to ameliorate suffering<br>• If anesthesia, euthanasia, or any kind of animal sacrifice is part of the study, include briefly which substances and/or methods were applied<br><br>**Field Research**<br><br>Include the following details if this study involves the collection of plant, animal, or other materials from a natural setting:<br>• Field permit number<br>• Name of the institution or relevant body that granted permission	
**Data Availability**<br><br>Authors are required to make all data underlying the findings described fully available, without restriction, and from the time of publication. PLOS allows rare exceptions to address legal and ethical concerns. See the PLOS Data Policy and FAQ for detailed information.	No - some restrictions will apply

A Data Availability Statement describing where the data can be found is required at submission. Your answers to this question constitute the Data Availability Statement and **will be published in the article**, if accepted.<br><br>**Important:** Stating 'data available on request from the author' is not sufficient. If your data are only available upon request, select 'No' for the first question and explain your exceptional situation in the text box.<br><br>Do the authors confirm that all data underlying the findings described in their manuscript are fully available without restriction?	
**Describe where the data may be found in full sentences. If you are copying our sample text, replace any instances of XXX with the appropriate details.**<br><br>• If the data are **held or will be held in a public repository**, include URLs, accession numbers or DOIs. If this information will only be available after acceptance, indicate this by ticking the box below. For example: *All XXX files are available from the XXX database (accession number(s) XXX, XXX.)*.<br>• If the data are all contained **within the manuscript and/or Supporting Information files**, enter the following: *All relevant data are within the manuscript and its Supporting Information files.*<br>• If neither of these applies but you are able to provide **details of access elsewhere**, with or without limitations, please do so. For example:<br><br>*Data cannot be shared publicly because of [XXX]. Data are available from the XXX Institutional Data Access / Ethics Committee (contact via XXX) for researchers who meet the criteria for access to confidential data.*<br><br>*The data underlying the results presented in the study are available from (include the name of the third party*	The data cannot be shared publicly due to ethical restrictions: this data was routinely collected from a marginalized population of undocumented refugees and migrants and the women have not consented for the data to be shared. These restrictions are in keeping with the policy of the Oxford Tropical Research Ethics Committee. However, de-identified data is available from the Mahidol-Oxford Research Unit institutional data access committee upon reasonable request from researchers who meet the criteria for access to confidential data (contact Rita Chanviriyavuth, email rita@tropmedres.ac).

and contact information or URL).<br>• This text is appropriate if the data are owned by a third party and authors do not have permission to share the data.<br><br>* typeset	
Additional data availability information:	

Manuscript
Click here to access/download;Manuscript;Manuscript_May_2025_clean_FIN

Optimal gestational weight gain and pregnancy outcomes, by BMI and height, in a marginalised population of women with short stature living along the Thailand-Myanmar border: a retrospective cohort, 2004-2023.

Mary Gouws[1], Rose McGready[1,2*], Wirichada Pan-ngum[1,3,4], Aung Myat Min[2], Nay Win Tun[2], Mary Ellen Gilder[1,2], Taco Jan Prins[5,6], Widi Yotyingaphiram[2], Mupawjay Pimanpanarak[2], Jacher Viladpai-Nguen[2], Nuttapol Panachuenwongsakul[2], François H Nosten[1,2] and Sue J Lee[1,4]

[1] Nuffield Department of Medicine, Centre for Tropical Medicine and Global Health, University of Oxford, Oxford, United Kingdom.

[2] Shoklo Malaria Research Unit, Mahidol-Oxford Tropical Medicine Research Unit, Faculty of Tropical Medicine, Mahidol University, Mae Sot, Thailand.

[3] Department of Tropical Hygiene, Faculty of Tropical Medicine, Mahidol University, Bangkok, Thailand.

[4] Mahidol-Oxford Tropical Medicine Research Unit, Faculty of Tropical Medicine, Mahidol University, Bangkok, Thailand.

[5] Department of Family Medicine, Faculty of Medicine, Chiang Mai University, Chiang Mai, Thailand.

[6] Amsterdam University Medical Centre, Department of Internal Medicine & Infectious Diseases, Research Groups: APH, GH and AII&I, Amsterdam UMC, Amsterdam, The Netherlands.

[*] Corresponding author

Email: rose@tropmedres.ac

Abstract

Background

Existing gestational weight gain (GWG) standards may not be applicable to women of short stature and those who have limited access to healthcare and are vulnerable to compromised nutrition during pregnancy. To inform the development of population-specific recommendations, this study investigated optimal GWG, by height and Body Mass Index (BMI), in a minority migrant and refugee population living along the Thailand-Myanmar border.

Methods

Records of all women attending antenatal care in the first trimester at the Shoklo-Malaria Research Unit between 2004 and 2023 were retrospectively examined. GWG of 17,194 women was assessed against maternal, delivery and neonatal outcomes, by height and per Asia-Pacific BMI category. The Gestation Related Optimal Weight centiles were used to classify small, appropriate and large for gestational age neonates. Multivariable logistic regression analysis, including natural cubic splines, was used to assess the relationships between GWG and outcomes of interest. Optimal GWG per BMI group was defined as the GWG associated with the lowest composite risk for adverse outcomes. The optimal range included GWG values that did not exceed a 5% increase from the corresponding minimum composite risk.

Results

Optimal GWG in women shorter than 153cm was lower per BMI group than the National Academy of Medicine and Intergrowth-21 recommendations: underweight 12.1kg (10.0-14.5), normal 10.4kg (8.0-12.9), overweight/obese 5.3kg (3.1-8.5); but comparable for women 153cm or more: underweight: 13.1kg (11.0–15.1), normal: 12.3kg (9.7-15.3), overweight/obese: 9.5kg (6.4-13.4).

Conclusion

Optimal GWG ranges are lower for this population with short stature compared to existing international guidelines. Clinical and contextual factors must be considered when implementing GWG recommendations for this, and other marginalized and short-stature populations.

Introduction

Weight gain during pregnancy is widely recognized as an important determinant of maternal, delivery and neonatal outcomes[1–4]. Excessive gestational weight gain (GWG) has been linked to adverse outcomes such as gestational diabetes mellitus, hypertensive disorders of pregnancy, preterm birth, caesarean section and neonates born large for gestational age. Inadequate GWG has been associated with preterm birth and small for gestational age [3,4].

The National Academy of Medicine (NAM), formerly known as the Institute of Medicine (IOM) produced GWG recommendations in 1990, which were updated in 2009, and used data from women in the United States of America and Western Europe to define optimal GWG[5–7]. However, a 2016 review showed that the NAM recommendations were derived from a combination of studies, between which there was significant heterogeneity in methodology and statistical analysis[8].

To produce GWG guidelines across a diverse group of countries, data was used from the Intergrowth-21 (IG-21) prospective cohort [6]. This study incorporated 4,607 women and used strict inclusion and exclusion criteria to define a healthy pregnancy and neonatal outcome to create GWG guidelines for women with a normal BMI of 18.5-24.9 kg/m^2 (based on WHO BMI classification) and a height of 153cm or more[6,9].

However, a 2021 study by Lee et al., in a marginalised group of women living along the Thailand-Myanmar found that short maternal stature was associated with lower GWG and suggested that height should be considered with first-trimester BMI when assessing ideal GWG during pregnancy[5]. This population has an average height of 151.5cm, with almost 60% of women below the IG-21 cut-off for height of 153cm[5]. Lee et al (2021) reported that appropriate for gestational age (AGA) in term newborns was achieved by 80% of women even though only 25% achieved GWG within NAM recommendations[5].

There is therefore a gap in the GWG literature for populations of short-stature who are at risk of compromised nutrition before and during pregnancy[5]. To inform the development of population-specific recommendations, this study investigated optimal GWG, by height and Body Mass Index (BMI), in a minority migrant and refugee population living along the Thailand-Myanmar border. The findings from our study may also be applicable to other populations of short stature globally, across socio-cultural contexts and healthcare services.

Methods

Ethical approval

Ethical approval was obtained through Oxford Tropical Research Ethics Committee (OxTREC reference 531-24) and the local body representing the community: Tak Province Community Advisory Board; T-CAB (TCAB202405). The T-CAB group is composed of community leaders who were consulted on the study design, process, and outcomes of interest[10].

Study setting

Shoklo Malaria Research Unit (SMRU), based in Mae Ramat, Thailand, is a field-based research organisation[11]. SMRU undertakes a combination of humanitarian clinical services and context-relevant research on both sides of Thailand-Myanmar border[12]. SMRU offers free antenatal, labour and delivery care to marginalised populations on the border[12]. SMRU served both refugee and migrant populations, however, healthcare services for refugee women were handed over to another humanitarian organisation in 2016. Thus, SMRU now supports Thailand's Public Health Department by filling a gap in care for migrant and displaced populations.

Study design

All pregnancy records from women with singleton pregnancies born at 28 weeks or later, between 01/01/2004 and 31/12/2023 were extracted from the SMRU electronic database. Antenatal and birth records are routinely collected and entered in a central electronic database which is securely managed by a local data team. The clinical information was gathered from medical documents that were originally maintained on paper and transitioned to electronic format in late 2008. Records on paper between 1986 and 2008 were digitized and incorporated into an electronic database to facilitate retrieval of antenatal care and birth outcome data. After 2008, all records have been entered directly into the electronic database.

The dataset for the purposes of this research was first accessed on 23/06/2024 and data extraction from the database was completed by 30/06/2024. When required for clarification, paper-based records were checked, which are stored at the SMRU offices. This was completed by 30/07/2024. All records were anonymised and identifiable information from the women was removed prior to analysis.

Participant inclusion/exclusion and study variables

A process of data extraction based on inclusion and exclusion criteria was followed. Only records of singleton pregnancies with an estimated gestational age (EGA) of less than 14 weeks at first antenatal visit, and at least two weight measurements over the course of the pregnancy were included. Women with last maternal weight recorded >4 weeks prior to delivery, pre-existing conditions such as hypertension, diabetes mellitus, HIV, moderate to severe anaemia at the first antenatal care visit and delivery prior to 28 weeks' gestation (spontaneous abortion) were excluded. Iatrogenic preterm births and neonates with major congenital abnormalities were also excluded. The small number of neonates with major congenital abnormalities were excluded due to their possible skewed birth weight distributions, such as being small or large for gestational age, which could confound the assessment of the relationship between maternal weight gain and neonatal outcomes. Additionally, certain congenital abnormalities can directly or indirectly influence GWG.

First trimester BMI (<14 weeks gestation) was used to represent pre-pregnancy BMI, as it has been shown to be an accurate approximation in several international cohorts[13–15]. The date of measurement of last maternal weight was used to calculate GWG and was required to be within four weeks of delivery to be included in the analysis. Trained midwives collected weight measurements at first ANC consultation and at each follow-up visit using mechanical Salter scales with 0.5kg precision. Gestational age was calculated by ultrasound, using crown-rump length between 9+0 to 13+6 weeks. The quality of scanning in this population has been confirmed and is reliable[16,17].

SGA, AGA and LGA classification was undertaken by calculating sex-, ethnicity- and gestational age-adjusted birthweight centiles using the Perinatal Institute's Gestation Related Optimal Weight (GROW) customised bulk centile calculator V.8.0[18]. GROW offers the advantage of region-specific classification and has coefficients to represent over 100 country-of-origin groups. Based on the average height, the Myanmar standards were used for women of Burmese ethnicity, and the Nepalese standards most closely matched the average height of Karen and other ethnicities in the dataset [19].

Neonates below the 10th percentile were classified as SGA, while those above the 90th percentile were classified as LGA[20,21]. Neonates at and between the 10th and 90th percentiles were classified as AGA.

Based on the literature and the availability of data in the cohort, the following maternal, birth and neonatal outcomes were considered for further analysis in relation to GWG: gestational diabetes mellitus (GDM), hypertensive disorders of pregnancy (HDoP), caesarean section (CS), preterm birth (birth <37 weeks' gestation), small-for gestational age (SGA) and large-for gestational age (LGA). HDoP included gestational hypertension, pre-eclampsia and eclampsia.

Statistical analysis

All statistical analyses were conducted using R (Version 4.2.1) and RStudio (Version 2024.04.0+735). Initial descriptive statistics were performed according to Asia-Pacific BMI categories which were defined as: underweight (<18.5 kg/m²), normal weight (18.5–22.9 kg/m²), overweight (23.0–24.9 kg/m²), and obese (≥25.0 kg/m²)[22,23]. For the purposes of this analysis, overweight and obese women were categorized into a single group as the narrow overweight range limited its utility as a distinct category and separating the overweight and obese groups resulted in small, underpowered groups.

Continuous variables with a normal distribution were summarised using mean ± standard deviation (SD), otherwise median and interquartile range (IQR; 25th-75th percentiles) were reported. Categorical variables were expressed as frequencies with percentages (n(%)). The chi-squared test and Cochran-Armitage test for trend were used to assess associations between categorical variables across BMI groups. Statistical significance was set at $p < 0.05$.

Univariable and multivariable logistic regression models were used to assess the relationships between GWG and outcomes. Multivariable models were adjusted for known confounders including age, parity, smoking status and ethnicity, based on the literature[3,24]. To address potential collinearity between age and parity, a categorisation to combine age and parity into six groups was created, and was used as a single categorical variable in the models. The groups were defined as age <20 and nulliparous; age <20 and parity ≥1; age 20-29 and nulliparous; age 20-29 and parity ≥1; age ≥ 30 and nulliparous; age ≥30 and parity ≥1.

Adjustment was also made for year (2004-2012, 2013-2016, 2017-2020, 2021-2023). This accounted for method of GDM screening, as this changed from no screening before 2013, to risk-factor based screening in 2013-2020, to full population screening after 2020[25] and also for the change in care provision to migrant populations only from 2017. Additionally, adjustment was made for malaria (treated during pregnancy), as it is a common infection in this area, and is associated with adverse outcomes including HDoP, SGA, preterm birth and stillbirth[16,26,27]. Although literacy and socioeconomic status are also possible confounders[4,24], reliable measures of these variables were not available within the dataset.

As visual inspection of the association between GWG and adverse outcomes indicated nonlinear relationships, natural cubic splines with logistic regression models were used. Natural cubic splines were chosen to mitigate overfitting, especially at the boundaries of the data.[28]. The splines were constructed as a series of polynomial functions, each defined within a specific interval of GWG. Each spline was joined smoothly using knots, ensuring that the overall function was continuous. The optimal number of knots was determined though visualisation of the relationship between predictor and outcome variables. All

models were constructed using 2 to 3 degrees of freedom (i.e. 3 to 4 knots) to maintain simplicity while capturing essential patterns.

Using these models, predicted probabilities were calculated for each adverse outcome across the range of GWG. For each weight, the predicted probabilities of all adverse outcomes were summed to derive a composite risk score. The minimum composite risk score was used to define the optimal GWG, with the optimal range corresponding to a composite score within 5% of the minimum risk. This methodology uses elements similar to those used by Choi et al. (2017) for a cohort of Korean women, by Morisaki et al. (2017) who considered GWG in Japanese women, and by Ee et al., in a Singaporean cohort (2014) [29–31].

Results

Fig 1 illustrates the selection process for the study cohort. From a total of 44,364 women with singleton pregnancies and deliveries at ≥28 weeks' gestation between January 1, 2004, and December 31, 2023, 25,565 women (58.0%) were excluded due to enrolment at ≥14 weeks' gestation (n=24,016) or incomplete height and weight data (n=1,549). Additionally, women with last maternal weight recorded >4 weeks prior to delivery (n=1,262), pre-existing conditions such as hypertension, diabetes mellitus, or HIV (n=75), moderate to severe anaemia at the first antenatal care visit (HCT <25%, n=48), iatrogenic preterm births (N=174) and neonates with major congenital abnormalities (n=46) were excluded. The final cohort analysed consisted of 17,194 women.

Figure 1. Flow of study participants.

Maternal demographic characteristics

Table 1 shows the characteristics, by Asia-Pacific BMI group, of the 17,194 women who met the inclusion criteria for the study between 2004 and 2023. More than half of women, 10,115 (58.8%) had a normal first trimester BMI, while 3,019 (17.6%) were underweight (minimum BMI 13.5 kg/m^2), and 4,060 (23.6%) overweight or obese by Asia-Pacific BMI standards (Table 1). There were 2089 women with an overweight BMI (23 - 24.9kg/m^2) and 1971 women with an obese (≥ 25kg/m^2) first trimester BMI (S1 Table).

In the underweight BMI group, the range was 4.9 kg/m² (13.5 to 18.4 kg/m²) and the range for the normal BMI category was bound at 4.4 kg/m² (18.5-22.9 kg/m²). In the overweight BMI group, the range was 1.9 kg/m² (23 - 24.9kg/m²), while for the obese group it was much wider, 18 kg/m² (25-43kg/m²). The proportion of women in the cohort with BMI ≥30 kg/m² (obese by WHO International Standards) was only 2% (356 of 17,194 women). The distribution of BMI can be seen in S1 Fig (supplementary).

The mean height (SD) for the cohort was 151.4cm (5.4cm), and more than half of women (10,205, 59.4%) had a height less than 153cm. The distribution of height in the cohort can be seen in the supporting information document (S2 Fig). Mean GWG for the full cohort was 9.3kg (SD 4.0kg), which decreased with increasing BMI category (test for trend $p<0.001$). Most women with a BMI <18.5kg/m² gained below NAM recommendations (2,266, 75.1%); only a quarter (696, 23.1%) gained within NAM guidelines. A similar pattern was present in the women with normal BMI, where 7,305 (72.2%) gained below NAM guidelines, while 2,363 (23.4%) gained within NAM recommendations, and 447 (4.4%) gained above NAM recommendations. In the overweight and obese group almost half (1,755, 43.2%) of women gained within NAM recommendations, with 1,154 (28.4%) gaining below and 1,151 (28.3%) gaining above NAM.

Most women had a vaginal birth (16,337 (95.0%)) while 857 (5.0%) delivered by caesarean section. Rates of caesarean section increased with increasing BMI category from 2.8% (underweight group) to 9.0% (overweight/obese group) (test for trend $p<0.001$).

Table 1. Maternal demographic and outcome descriptive statistics by Asia-Pacific BMI categories.

Variable	All Women, n (%)	Underweight (<18.5 kg/m²)	Normal (18.5–22.9 kg/m²)	Overweight & Obese (≥23 kg/m²)
**Total Women**	17,194	3,019 (17.6)	10,115 (58.8)	4,060 (23.7)
**Median Age (IQR), years**	25 (20–30)	23 (20–28)	24 (20–30)	28 (23–33)
**Nulliparous (%)**	6231 (36.2)	1345 (44.6)	3933 (38.9)	953 (23.5)
**Ethnicity**				
Karen (%)	8,994 (52.3)	1,383 (45.8)	5,427 (53.7)	2,184 (53.8)
Burmese (%)	4,160 (24.2)	884 (29.3)	2,154 (21.3)	1,122 (27.6)
Other (%)	4,040 (23.5)	752 (24.9)	2,534 (25.1)	754 (18.6)
**Mean Height (SD), cm**	151.4 (5.4)	152 (5.5)	151.1 (5.4)	151.8 (5.4)
**Height <153 cm (%)**	10,205 (59.4)	1,692 (56.0)	6,198 (61.3)	2,315 (57.0)

**Mean GWG (SD), kg**	9.3 (4.0)	10.2 (3.5)	9.5 (3.8)	8.2 (4.4)
**NAM[a] GWG Recommendation (kg)**				
Below NAM (%)	10,725 (62.4)	2,266 (75.1)	7,305 (72.2)	1,154 (28.4)
Within NAM (%)	4,814 (28.0)	696 (23.1)	2,363 (23.4)	1,755 (43.2)
Above NAM (%)	1,655 (9.6)	57 (1.9)	447 (4.4)	1,151 (28.3)
**Smokers (%)[b]**	2,650 (15.4)	588 (18.5)	1,673 (16.5)	419 (10.3)
**Hypertensive Disorder (%)**	1,127 (6.6)	110 (3.6)	557 (5.5)	460 (11.3)
**Gestational Diabetes Mellitus (%)[c]**	628 (3.7)	77 (2.6)	219 (2.2)	332 (8.2)
**Malaria in Pregnancy (%)**	1,703 (9.9)	385 (12.8)	1,077 (10.6)	241 (5.9)
**Caesarean Section (%)**	857 (5.0)	86 (2.8)	405 (4.0)	366 (9.0)

[a]NAM = National Academy of Medicine. [b]Gestational Diabetes Mellitus, n=7 values missing. [c]Smoking, n=47 values missing.

Delivery and neonatal outcomes

Birth outcome was available for all 17,194 neonates (Fig 1), of which 17,109 (99.5%) were live births (Table 2). Of all neonates, 1,048 (6.1%) were born preterm. Birthweight for gestational age centiles were calculated for 15,783 (91.8%) neonates who had a birthweight measured within 72 hours of delivery. While most babies were born AGA (12,402 (78.6%)), nearly one in six were SGA (2,463 (15.6%)), and a minority, one in twenty, (918 (5.8%)) were LGA.

Table 2. Neonatal descriptive statistics by maternal Asia-Pacific BMI categories.

**Variable**	**All**	**Underweight (<18.5 kg/m²)**	**Normal (18.5–22.9 kg/m²)**	**Overweight & Obese (≥23 kg/m²)**
**All births with known outcome (%)**	17,194	3,019 (17.6)	10,115 (58.8)	4,060 (23.6)
Live births	17,109 (99.5)	2.999 (99.3)	10,065 (99.5)	4,045 (99.6)
Stillbirths	85 (0.5)	20 (0.7)	50 (0.5)	15 (0.4)
Male[a]	8,648 (50.3)	1,494 (49.5)	5,096 (50.4)	2,058 (50.7)
Median EGA (IQR), weeks	39.3 (38.4–40.1)	39.2 (38.3–40.0)	39.3 (38.4–40.1)	39.4 (38.5–40.2)
Preterm births <37 weeks (%)	1,048 (6.1)	259 (8.6)	645 (6.4)	144 (3.5)
**Birthweight measured <72 hours[b]**	15,783	2,672 (16.9)	9,284 (58.8)	3,827 (24.2)
Mean Birthweight (SD), g	2,982.0 (446.8)	2,843.5 (440.9)	2,959.0 (434.4)	3,134.6 (438.3)

SGA (%)	2,463 (15.6)	470 (17.6)	1,414 (15.2)	579 (15.1)
AGA (%)	12,402 (78.6)	2,061 (77.1)	7,333 (79.0)	3,008 (78.6)
LGA (%)	918 (5.8)	141 (5.3)	537 (5.8)	240 (6.3)

[a]Male baby, n=6 values missing.

[b]Birthweight for gestational age centiles were calculated for 15,783 (91.8%) neonates who had a birthweight measured within 72 hours of delivery

Calculation of optimal GWG

Two thirds of the pregnancies did not experience HDoP, GDM, CS, spontaneous preterm birth, SGA or LGA (11,309 women (65.8%)), 4,878 (28.4%) had one adverse outcome, and less than 6% experienced two or more adverse outcomes (1,007 women (5.6%)). The six outcomes of HDoP, GDM, CS, spontaneous preterm birth, SGA and LGA were plotted as predicted probabilities against GWG using smoothed restricted cubic splines for the BMI categories of underweight, normal and overweight/obese, each for two height categories of <153 and ≥ 153cm (Figs 2-4). The GWG at which the composite risk score for adverse outcomes was lowest was used to calculate an optimal GWG, and range (Table 3).

In the cohort of women with an underweight BMI in the first trimester and height <153cm, the optimal GWG was 12.1 (10.0-14.5kg), while in the underweight group with height of 153cm or more, the optimal GWG was higher, at 13.1kg (11.0-15.1kg) (Fig 2). A similar pattern was seen across both the normal BMI and overweight/obese groups, in which the women with a height <153cm had a lower optimal GWG than the taller women (Figs 3 & 4). In the women with a normal first trimester BMI, optimal GWG in the <153cm cohort was 10.4kg (8.0-12.9kg), while for normal BMI women with height ≥153cm, optimal GWG was 12.3kg (9.7-15.3kg) (Fig 3). In the combined overweight/obese first-trimester BMI group, in the women with height <153cm, optimal GWG was 5.3kg (3.1-8.5kg), while in those ≥153cm, optimal GWG was 9.5kg (6.4-13.4kg) (Fig 4). S3, S4 and S5 Figs in the supporting information show the calculated optimal GWG ranges superimposed on the predicted probabilities of adverse maternal and neonatal outcomes, stratified by BMI and height categories.

Figure 2. Predicted probabilities with 95% confidence intervals (y-axis) of adverse outcomes across GWG (x-axis) for women with an underweight first trimester BMI. Calculated using logistic regression models incorporating cubic splines. NAM GWG recommendations superimposed as vertical grey lines at 12.5 and 18.0kg (a) Women with underweight BMI and height <153cm, N=1,692; (b) Women with underweight BMI and height ≥153cm, N= 1,327.
Key: CS=Caesarean Section; GDM=Gestational Diabetes Mellitus; HDoP=Hypertensive Disorders of Pregnancy; LGA=Large for Gestational Age; Preterm=Preterm birth; SGA=Small for Gestational Age.

Figure 3. Predicted probabilities with 95% confidence intervals (y) of adverse outcomes across GWG (x) for women with a normal first trimester BMI. Calculated using logistic regression models incorporating cubic splines. NAM GWG

Asia-Pacific BMI category (kg/m²)	Optimal GWG (range), kg full cohort	Optimal GWG (range), kg height <153cm	Optimal GWG (range), kg height ≥153cm	NAM GWG recommendation, kg[a]
Underweight (<18.5)	15.1 (12.4 – 18.6) N=3,038	12.1 (9.9-14.5) N=1,706	13.5 (11.5-15.4) N=1,332	12.5-18.0
Normal (18.5-22.9)	12.6 (9.6 – 15.6) N=10,213	11.9 (9.2-14.3) N=6,262	13.3 (10.3-16.3) N=3,951	11.5-16.0
Overweight/Obese (≥23)	7.7 (4.9-11.8) N=4,117	8.5 (5.2-11.3) N=2,350	10.8 (8.04-16.0) N=1,767	Overweight: 7-11.50 Obese: 5.0-9.0

recommendations superimposed as vertical grey lines at 11.5 and 16.0kg (a) Women with normal BMI and height <153cm, N=6,198; (b) Women with normal BMI and height ≥153cm, N=3,917.

Figure 4. Predicted probabilities with 95% confidence intervals (y) of adverse outcomes across GWG (x) for women with an overweight or obese first trimester BMI. Calculated using logistic regression models incorporating cubic splines. NAM GWG recommendations superimposed as vertical grey lines at 7.0 and 11.5kg (a) Women with overweight or obese BMI and height <153cm, N=2,315; (b) Women with overweight or obese BMI and height ≥153cm, N=1,745.

Table 3. Optimal GWG ranges based on minimisation of composite risk of adverse outcomes per BMI category.

Asia-Pacific BMI category (kg/m²)	Optimal GWG (range), kg full cohort	Optimal GWG (range), kg height <153cm	Optimal GWG (range), kg height ≥153cm	NAM GWG recommendation, kg[a]
Underweight (<18.5)	12.4 (10.2– 14.8) N=3,019	12.1 (10.0-14.5) N=1,692	13.1 (11.0-15.1) N=1,327	12.5-18.0
Normal (18.5-22.9)	11.0 (8.3 – 14.0) N=10,115	10.4 (8.0-12.9) N=6,198	12.3 (9.7-15.3) N=3,917	11.5-16.0
Overweight/Obese (≥23)	9.0 (5.6-12.1) N=4,060	5.3 (3.1-8.5) N=2,315	9.5 (6.4-13.4) N=1,745	Overweight: 7-11.5 Obese: 5.0-9.0

[a]For reference: NAM GWG recommendations are based on WHO BMI classifications which are defined as follows: underweight (<18.5 kg/m²), normal (18.5–24.9 kg/m²), overweight (25.0–29.9 kg/m²), obese (≥30.0 kg/m²).

Discussion

GWG ranges based on first-trimester Asia-Pacific BMI categories were calculated to minimize the risks of adverse pregnancy and birth outcomes. In general, the GWG ranges for the migrant and refugee population were lower than the NAM guidelines. For women with height less than 153cm and normal BMI, the optimal GWG was 10.4kg with range 8.0-12.9 kg compared with 11.5-16.0 from the NAM guidelines.

The NAM guidelines, primarily derived from women in North America (average height of 162 cm) and Western Europe (166 cm), recommend a GWG of 11.5-16kg at term in normal BMI women by WHO BMI standards[7,19]. IG-21, which included only women with a height of 153cm or more, provide a 50th centile GWG recommendation of 13.2kg (10th-90th centiles: 8.3-19.4kg) for normal BMI women (by WHO BMI standards) at 39 weeks' gestation (equivalent to the mean gestation at delivery in the migrant and refugee cohort)[6]. In this analysis, when restricted to women of 153cm or more, optimal GWG (12.3kg, range: 9.7-15.3) was comparable to the IG-21 range. However, for women shorter than 153cm, the optimal GWG was lower (10.4kg, range: 8.0-12.9). For the underweight BMI category, in which NAM recommends a GWG of 12.5-18kg, in this analysis optimal GWG was 13.1kg (11.0-15.1) in women ≥153cm, while in underweight women <153cm, optimal GWG was lower: 12.1kg (10.0-14.5).

Morisaki et al. (2017) included 104 070 Japanese women with singleton pregnancies to find the GWG with lowest risk of adverse outcomes (SGA, preterm birth, pre-eclampsia and complicated delivery: CS, forceps or vacuum delivery, obstructed labour, PPH)[30]. Morisaki et al. calculated the optimal weight gain to minimise adverse outcomes, by BMI groups (kg/m^2): BMI 17.0–18.4: 12.2 kg; BMI 18.5–19.9: 10.9kg; BMI 20–22.9: 9.9kg; 23–24.9: 7.7kg; and 25–27.4: 4.3kg[30]. This Japanese population had a mean height of 158cm (lower than the average for American and European populations) and the optimal GWG ranges were close to those developed for the cohort of women with short stature anaylsed here[30].

An additional finding in the current analysis of women living along the Thailand-Myanmar border is that the proportion of women with BMI ≥30 kg/m^2 is only 2%, which contrasts with countries such as the US, where this proportion is estimated to be higher than 20%[32,33]. In the Thai-Myanmar border population, where only a small proportion of women fall into the overweight/obese category, the overall GWG distribution may be skewed by the higher proportion of women at the lower end of the BMI spectrum. This finding highlights the role that BMI distribution plays in shaping GWG patterns within a population and may also explain why the interval for GWG in the women with first trimester overweight and obese BMI was wider than that calculated for normal and underweight BMI groups (Table 3). This wider interval can be attributed to the wide variation of first trimester BMI in the overweight and obese group, which ranged from 23 to 43kg/m^2. In the underweight BMI group, the range was only 4.9 kg/m^2 (13.5 to 18.4 kg/m^2) and the range for the normal BMI category was bound at 4.4 kg/m^2

(18.5-22.9 kg/m²). Thus, applying guidelines developed in populations with different BMI distributions may not fully capture the nuanced needs and unique demographic and health characteristics of all populations.

This analysis also had other limitations. While in this study, the optimal GWG and ranges have been calculated, the pattern and timing of weight gain across gestation is equally, if not more, important[34]. Longitudinal data is crucial for understanding the impact of GWG during different trimesters, and to account for changes in maternal and foetal development across various stages of pregnancy[34]. Furthermore, data on literacy and socioeconomic status, which can influence health and well-being of pregnant women and affect the GWG, was limited for this cohort, thus could not be adjusted for in the analysis. However, we do know that all women in this cohort were seeking services in a humanitarian healthcare setting.

We were unable to adjust for within subject correlation due to repeat pregnancies (i.e., mothers being included in the dataset more than once). Data collection for this was only available for more recent pregnancies and as data collection continues, this is an aspect of analysis that will be included in future studies.

An additional limitation to the approach used for calculating optimal GWG in this analysis is that the composite scores are likely overestimating risk because the interrelated nature of certain adverse outcomes is not accounted for. For instance, GDM, CS and LGA may co-occur and the calculation of the composite score which considers each of these as separate risks might overestimate the total calculated risk. However, in this cohort the proportion of women with two or more adverse outcomes was less than 6%, thus, the impact of "overestimating" risk was likely minor. Instead, the GWG ranges reported here would be most strongly influenced by the two thirds of women who had no adverse events.

Additionally, the screening and diagnosis of GDM has changed over time in this population, and thus the estimation of risk for GDM may not be representative of the true rates in this population. Future analyses should consider an approach to account for the potential overlap between adverse outcomes to provide a more accurate risk assessment.

To get an accurate reflection of GWG patterns and classify women into BMI groups, the data in this analysis was limited to women who had their first antenatal care (ANC) visit before 14 weeks' gestation. This process excluded approximately 40% of women who gave birth between 2004 and 2023, reflective of the real-world situation where many women do not access care early in gestation or do not have a documented first trimester BMI. If, for example, a woman at 20 weeks' estimated gestational age presents for a first time ANC visit, already having gained weight, it would be unclear which recommendations should be followed. Additionally, classifying BMI, a continuous variable, into groups comes at the expense of classifying women with a BMI of 22.8 and 23.0 as more different than similar, and then applying differing GWG

recommendations to these women. Likewise, the height categories applied in this analysis were broad and may lead to recommendations that do not apply well to rare outliers (<140 cm or >160 cm) in this population.

A third challenge of BMI categorisation was the sample sizes per BMI group. The relatively smaller number of women with overweight (N=2089) and obese (N=1971) first-trimester BMIs meant that when the analysis was run for these groups individually the results were underpowered. Hence overweight and obese were combined for the analysis although overweight and obese women are not a homogenous group and ideally these groups would be analysed separately.

To address the limitations of BMI categorisation, further research should focus on the development of an interactive tool into which height, current weight and gestation can be entered, with an output of optimal GWG based on these criteria. Alternatively, methods of estimating GWG which do not rely on first trimester or pre-pregnancy BMI could also be considered. One example of this is mid-upper arm circumference (MUAC) measurement, which was proven to be strongly associated with first-trimester BMI and GWG in Darling et al.'s 2023 systematic review and meta-analysis [24]. Future studies could consider how MUAC changes over gestation and if MUAC can be used to accurately represent BMI or GWG patterns, especially in women who do not enter antenatal care in first trimester.

Conclusion

This study contributes to the growing body of literature on GWG by focusing on a unique and marginalized population of women of short-stature living along the Thailand-Myanmar border. The international NAM guidelines, based on taller populations from North America and Western Europe, overestimate the optimal GWG for this migrant and refugee cohort, which has an average height of 151.4cm. The GWG ranges developed in this study can be applied in clinical practice when advising women attending antenatal care at SMRU on the amount of weight to gain during pregnancy. There are limited data internationally on pregnant women with short stature globally, thus the findings from our study may also have applicability to other populations of short stature worldwide.

Supporting information

S1 File. Figure S1-S5. Table S1-3.

(PDF)

Acknowledgments

We recognize the efforts of the maternal and child health team at SMRU whose commitment to patient care and data collection has been foundational to this research and represents the collective work of many people over many years.

Author contributions

Conceptualization: Mary Gouws, Sue J. Lee, Wirichada Pan-ngum, Rose McGready.

Methodology: Mary Gouws, Sue J. Lee.

Data curation: Aung Myat Min, Nay Win Tun, Mary Ellen Gilder, Taco Jan Prins, Widi Yotyingaphiram, Mupawjay Pimanpanarak, Jacher Viladpai-Nguen, Nuttapol Panachuenwongsakul, François H Nosten, Rose McGready.

Formal analysis: Mary Gouws.

Supervision: Sue J. Lee, Wirichada Pan-ngum, Rose McGready.

Writing – original draft: Mary Gouws.

Writing – review & editing: Sue J. Lee, Wirichada Pan-ngum, Aung Myat Min, Nay Win Tun, Mary Ellen Gilder, Taco Jan Prins, Widi Yotyingaphiram, Mupawjay Pimanpanarak, Jacher Viladpai-Nguen, Nuttapol Panachuenwongsakul, François H Nosten, Rose McGready.

References

1. Crane JMG, White J, Murphy P, Burrage L, Hutchens D. The Effect of Gestational Weight Gain by Body Mass Index on Maternal and Neonatal Outcomes. Journal of Obstetrics and Gynaecology Canada. 2009;31: 28–35. doi:10.1016/S1701-2163(16)34050-6

2. LifeCycle Project-Maternal Obesity and Childhood Outcomes Study Group. Association of Gestational Weight Gain With Adverse Maternal and Infant Outcomes. JAMA. 2019;321: 1702–1715. doi:10.1001/jama.2019.3820

3. Goldstein RF, Abell SK, Ranasinha S, Misso M, Boyle JA, Black MH, et al. Association of Gestational Weight Gain With Maternal and Infant Outcomes: A Systematic Review and Meta-analysis. JAMA. 2017;317: 2207–2225. doi:10.1001/jama.2017.3635

4. Goldstein RF, Abell SK, Ranasinha S, Misso ML, Boyle JA, Harrison CL, et al. Gestational weight gain across continents and ethnicity: systematic review and meta-analysis of maternal and infant outcomes in more than one million women. BMC Med. 2018;16: 153. doi:10.1186/s12916-018-1128-1

5. Lee SJ, Hashmi AH, Min AM, Gilder ME, Tun NW, Wah LL, et al. Short maternal stature and gestational weight gain among refugee and migrant women birthing appropriate for gestational age term newborns: a retrospective cohort on the Myanmar-Thailand border, 2004-2016. BMJ Glob Health. 2021;6: e004325. doi:10.1136/bmjgh-2020-004325

6. Cheikh Ismail L, Bishop DC, Pang R, Ohuma EO, Kac G, Abrams B, et al. Gestational weight gain standards based on women enrolled in the Fetal Growth Longitudinal Study of the INTERGROWTH-21st Project: a prospective longitudinal cohort study. BMJ. 2016;352: i555. doi:10.1136/bmj.i555

7. Institute of Medicine (US) and National Research Council (US) Committee to Reexamine IOM Pregnancy Weight Guidelines. Weight Gain During Pregnancy: Reexamining the Guidelines. Rasmussen KM, Yaktine AL, editors. Washington (DC): National Academies Press (US); 2009. Available: http://www.ncbi.nlm.nih.gov/books/NBK32813/

8. Ohadike CO, Cheikh-Ismail L, Ohuma EO, Giuliani F, Bishop D, Kac G, et al. Systematic Review of the Methodological Quality of Studies Aimed at Creating Gestational Weight Gain Charts. Advances in Nutrition. 2016;7: 313–322. doi:10.3945/an.115.010413

9. Gestational Weight Gain | Intergrowth. [cited 3 Aug 2024]. Available: https://intergrowth21.com/tools-resources/gestational-weight-gain

10. Lwin KM, Peto TJ, White NJ, Day NPJ, Nosten F, Parker M, et al. The practicality and sustainability of a community advisory board at a large medical research unit on the Thai-Myanmar border. Health (Irvine Calif). 2013;5: 229–236. doi:10.4236/health.2013.52031

11. SMRU Clinics | Shoklo Malaria Research Unit. [cited 8 May 2024]. Available: https://www.shoklo-unit.com/humanitarian-activities/smru-clinics

12. The overlap between miscarriage and extreme preterm birth in a limited-resource setting on the Thailand-Myanmar border: a population cohort study - PMC. [cited 17 May 2024]. Available: https://www.ncbi.nlm.nih.gov/pmc/articles/PMC6305214/

13. Rangel Bousquet Carrilho T, M. Rasmussen K, Rodrigues Farias D, Freitas Costa NC, Araújo Batalha M, E. Reichenheim M, et al. Agreement between self-reported pre-pregnancy weight and measured first-trimester weight in Brazilian women. BMC Pregnancy Childbirth. 2020;20: 734. doi:10.1186/s12884-020-03354-4

14. Inskip H, Crozier S, Baird J, Hammond J, Robinson S, Cooper C, et al. Measured weight in early pregnancy is a valid method for estimating pre-pregnancy weight. Journal of Developmental Origins of Health and Disease. 2021;12: 561–569. doi:10.1017/S2040174420000926

15. Natamba BK, Sanchez SE, Gelaye B, Williams MA. Concordance between self-reported pre-pregnancy body mass index (BMI) and BMI measured at the first prenatal study contact. BMC Pregnancy Childbirth. 2016;16: 187. doi:10.1186/s12884-016-0983-z

16. Rijken MJ, Mulder EJH, Papageorghiou AT, Thiptharakun S, Wah N, Paw TK, et al. Quality of ultrasound biometry obtained by local health workers in a refugee camp on the Thai-Burmese border. Ultrasound Obstet Gynecol. 2012;40: 151–157. doi:10.1002/uog.11091

17. Rijken MJ, Lee SJ, Boel ME, Papageorghiou AT, Visser GHA, Dwell SLM, et al. Obstetric ultrasound scanning by local health workers in a refugee camp on the Thai–Burmese border. Ultrasound in Obstetrics & Gynecology. 2009;34: 395–403. doi:10.1002/uog.7350

18. Gestation Network, Perinatal Institute. Gestation Related Optimal Weight: Global Bulk Centile Calculator. [cited 18 Jun 2024]. Available: https://www.gestation.net/cc/about.htm

19. Average height for men and women worldwide. In: Worlddata.info [Internet]. [cited 13 May 2024]. Available: https://www.worlddata.info/average-bodyheight.php

20. Battaglia FC, Lubchenco LO. A practical classification of newborn infants by weight and gestational age. J Pediatr. 1967;71: 159–163. doi:10.1016/s0022-3476(67)80066-0

21. Chew LC, Verma RP. Fetal Growth Restriction. StatPearls. Treasure Island (FL): StatPearls Publishing; 2024. Available: http://www.ncbi.nlm.nih.gov/books/NBK562268/

22. World Health Orgnaisation, International Diabetes Institute. The Asia-Pacific Perspective: Redefining Obesity and its Treatment. World Health Organisation; 2020 Feb. Available: https://iris.who.int/bitstream/handle/10665/206936/0957708211_eng.pdf

23. Tham KW, Abdul Ghani R, Cua SC, Deerochanawong C, Fojas M, Hocking S, et al. Obesity in South and Southeast Asia—A new consensus on care and management. Obesity Reviews. 2023;24: e13520. doi:10.1111/obr.13520

24. Darling AM, Wang D, Perumal N, Liu E, Wang M, Ahmed T, et al. Risk factors for inadequate and excessive gestational weight gain in 25 low- and middle-income countries: An individual-level participant meta-analysis. PLOS Medicine. 2023;20: e1004236. doi:10.1371/journal.pmed.1004236

25. Prüst JT, Brummaier T, Wah M, Yee HH, Win NN, Pimanpanarak M, et al. Risk factor-based screening compared to universal screening for gestational diabetes mellitus in marginalized Burman and Karen populations on the Thailand-Myanmar border: An observational cohort. Wellcome Open Res. 2022;7: 132. doi:10.12688/wellcomeopenres.17743.2

26. Moore KA, Simpson JA, Scoullar MJL, McGready R, Fowkes FJI. Quantification of the association between malaria in pregnancy and stillbirth: a systematic review and meta-analysis. The Lancet Global Health. 2017;5: e1101–e1112. doi:10.1016/S2214-109X(17)30340-6

27. Harrington WE, Moore KA, Min AM, Gilder ME, Tun NW, Paw MK, et al. Falciparum but not vivax malaria increases the risk of hypertensive disorders of pregnancy in women followed prospectively from the first trimester. BMC Med. 2021;19: 98. doi:10.1186/s12916-021-01960-3

28. Gauthier J, Wu QV, Gooley TA. Cubic splines to model relationships between continuous variables and outcomes: a guide for clinicians. Bone Marrow Transplant. 2020;55: 675–680. doi:10.1038/s41409-019-0679-x

29. Choi SK, Lee G, Kim YH, Park IY, Ko HS, Shin JC. Determining optimal gestational weight gain in the Korean population: a retrospective cohort study. Reprod Biol Endocrinol. 2017;15: 67. doi:10.1186/s12958-017-0280-3

30. Morisaki N, Nagata C, Jwa SC, Sago H, Saito S, Oken E, et al. Pre-pregnancy BMI-specific optimal gestational weight gain for women in Japan. J Epidemiol. 2017;27: 492–498. doi:10.1016/j.je.2016.09.013

31. Ee TX, Allen Jr JC, Malhotra R, Koh H, Østbye T, Tan TC. Determining optimal gestational weight gain in a multiethnic Asian population. Journal of Obstetrics and Gynaecology Research. 2014;40: 1002–1008. doi:10.1111/jog.12307

32. Wang Y, Beydoun MA, Min J, Xue H, Kaminsky LA, Cheskin LJ. Has the prevalence of overweight, obesity and central obesity levelled off in the United States? Trends, patterns, disparities, and future projections for the obesity epidemic. International Journal of Epidemiology. 2020;49: 810–823. doi:10.1093/ije/dyz273

33. Wang MC, Freaney PM, Perak AM, Greenland P, Lloyd-Jones DM, Grobman WA, et al. Trends in Prepregnancy Obesity and Association With Adverse Pregnancy Outcomes in the United States, 2013 to 2018. Journal of the American Heart Association: Cardiovascular and Cerebrovascular Disease. 2021;10: e020717. doi:10.1161/JAHA.120.020717

34. Liang H, Yin C, Dong X, Acharya G, Li X. Clusters of week-specific maternal gestational weight gain pattern and their association with birthweight: an observational cohort study. Acta Obstetricia et Gynecologica Scandinavica. 2017;96: 1251–1260. doi:10.1111/aogs.13204

Fig 3

Click here to access/download;Figure;Fig3.tif

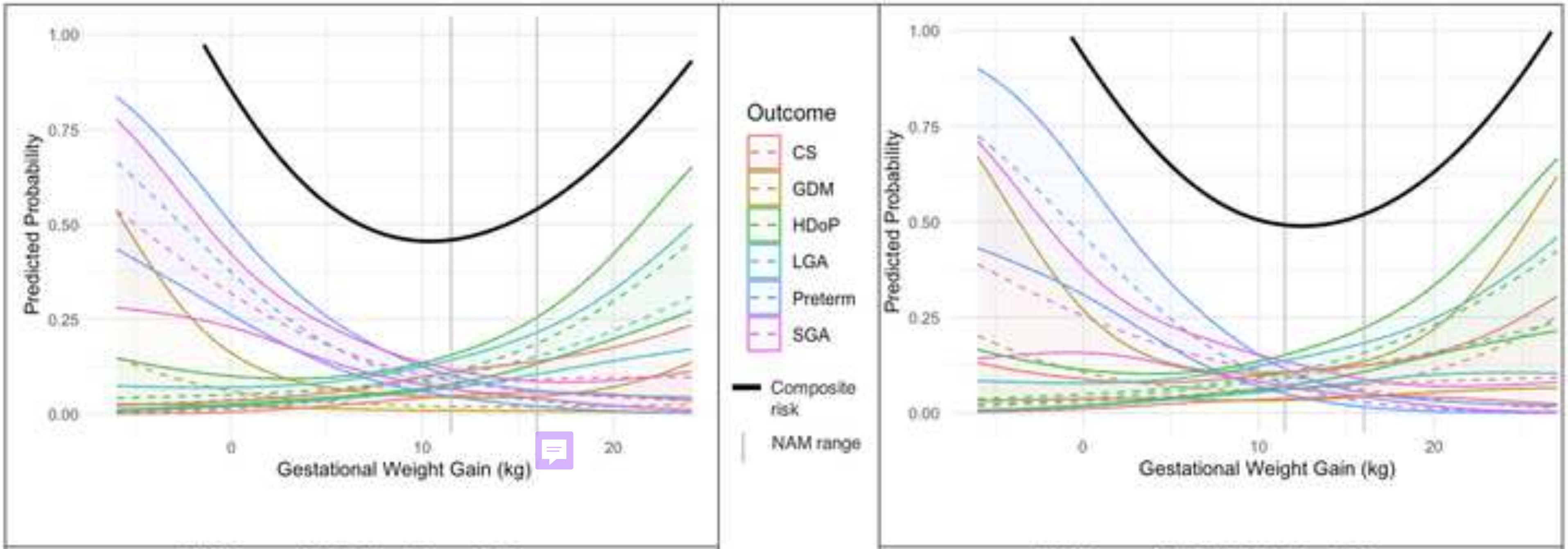

(a) Normal BMI, height <153cm

(b) Normal BMI, height ≥153cm

Fig 1

Click here to access/download;Figure;Fig1.tif

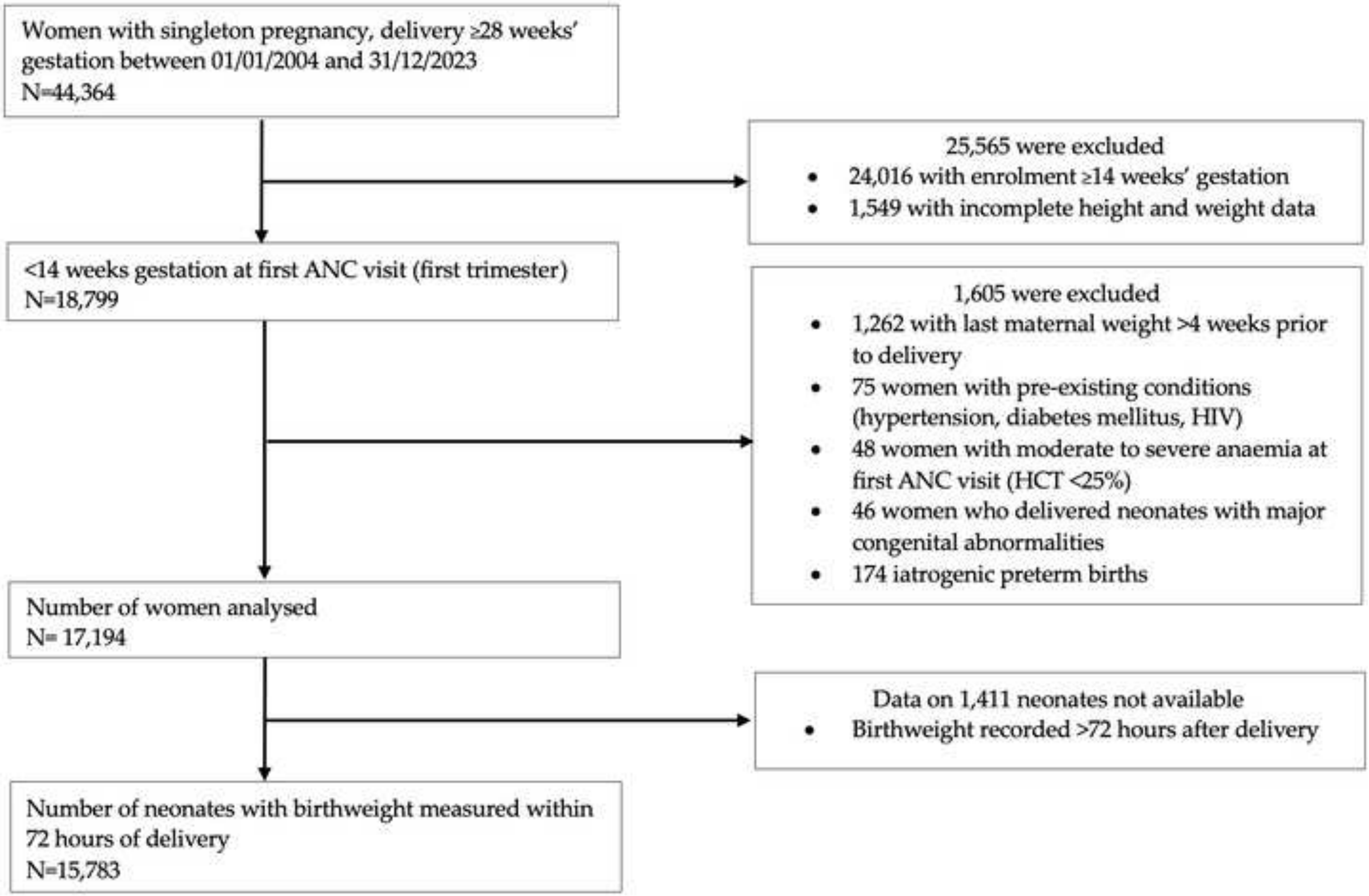

Fig 2

Click here to access/download;Figure;Fig2.tif

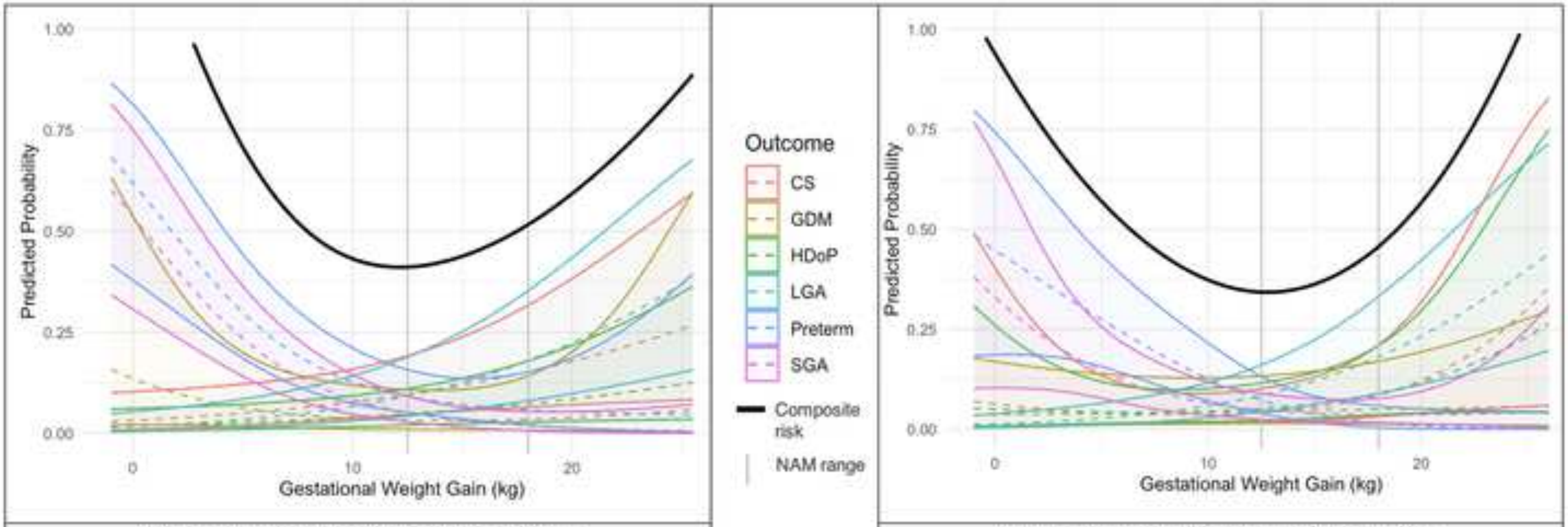

(a) Underweight BMI, height <153cm

(b) Underweight BMI, height ≥153cm

 Click here to access/download;Figure;Fig4.tif

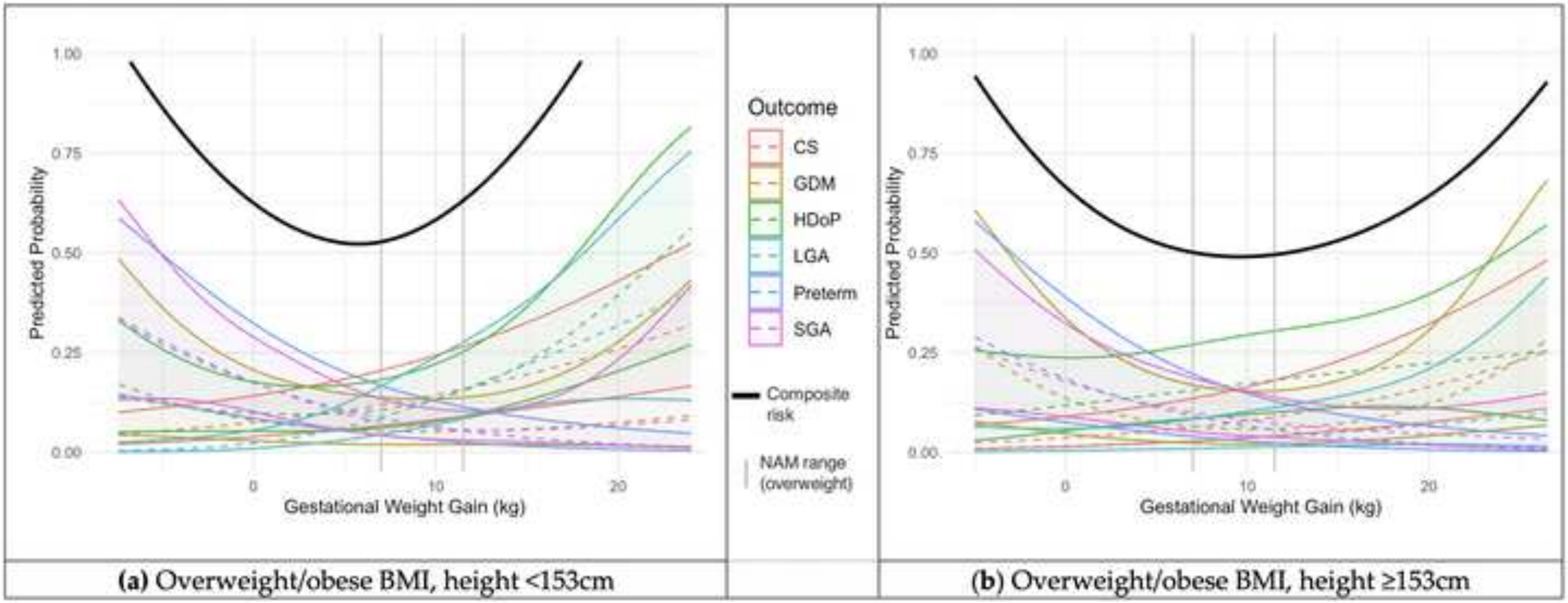

Click here to access/download
Supporting Information
Supplementary file.pdf

Optimal gestational weight gain and pregnancy outcomes, by BMI and height, in a marginalised ~~short stature~~ population of women with short stature living along the Thailand-Myanmar border: a retrospective cohort, 2004-2023.

Mary Gouws[1], ~~Sue J Lee[1,2]~~ Rose McGready[1,2*~~4~~], Wirichada Pan-ngum[1,3~~2~~,4~~3~~], Aung Myat Min[2~~4~~], Nay Win Tun[2~~4~~], Mary Ellen Gilder[1,2~~4~~], Taco Jan Prins[5,6], Widi Yotyingaphiram[2~~4~~], Mupawjay Pimanpanarak[2~~4~~], Jacher Viladpai-Nguen[2~~4~~], Nuttapol Panachuenwongsakul[2~~4~~], François H Nosten[1,~~2~~4] and Sue J Lee[1,4~~2~~] ~~Rose McGready[1,4]~~

[1] Nuffield Department of Medicine, Centre for Tropical Medicine and Global Health, University of Oxford, Oxford, United Kingdom.

[2] Shoklo Malaria Research Unit, Mahidol-Oxford Tropical Medicine Research Unit, Faculty of Tropical Medicine, Mahidol University, Mae Sot, Thailand.

~~Mahidol-Oxford Tropical Medicine Research Unit, Faculty of Tropical Medicine, Mahidol University, Bangkok, Thailand.~~

[3] Department of Tropical Hygiene, Faculty of Tropical Medicine, Mahidol University, Bangkok, Thailand.

[4] Mahidol-Oxford Tropical Medicine Research Unit, Faculty of Tropical Medicine, Mahidol University, Bangkok, Thailand.

~~Shoklo Malaria Research Unit, Mahidol-Oxford Tropical Medicine Research Unit, Faculty of Tropical Medicine, Mahidol University, Mae Sot, Thailand.~~

[5] Department of Family Medicine, Faculty of Medicine, Chiang Mai University, Chiang Mai, Thailand.

[6] Amsterdam University Medical Centre, Department of Internal Medicine & Infectious Diseases, Research Groups: APH, GH and AII&I, Amsterdam UMC, Amsterdam, The Netherlands.

[*] Corresponding author

Email: ~~mary.gouws@ndm.ox.ac.uk~~ Sue@tropmedres.ac

Abstract

Background

Existing gestational weight gain (GWG) standards may not be applicable to marginalised or short stature women of short stature and those who have limited access to healthcare and are vulnerable to compromised nutrition during pregnancy. To inform the development of population-specific recommendations, this study investigateds optimal GWG, by height and Body Mass Index (BMI), in a minority migrant and refugee population living along the Thailand-Myanmar border.

Methods

Records of all migrant women attending antenatal care in the first trimester at the Shoklo-Malaria Research Unit between 2004 and 2023 were retrospectively examined. GWG of 17,194368 women was assessed against maternal, delivery and neonatal outcomes, by height and per Asia-Pacific BMI category. The Gestation Related Optimal Weight centiles were used to classify small, appropriate and large for gestational age neonates. Multivariable logistic regression analysis, including natural cubic splines, was used to assess the relationships between GWG and outcomes of interest. Optimal GWG per BMI group was defined as the GWG associated with the lowest composite risk for adverse outcomes. The optimal range included GWG values that did not exceed a 5% increase from the corresponding minimum composite risk.

Results

Optimal GWG in women shorter than 153cm was lower per BMI group than the National Academy of Medicine and Intergrowth-21 recommendations: underweight 12.11kg (10.09.9-14.54.5), normal 10.48.9kg (8.07.1-12.91.3), overweight/obese 5.36.9kg (3.17-8.59.7); but comparable for women 153cm or more: underweight: 13.15kg (11.05–15.14), normal: 123.30kg (9.710.3-15.36.0), overweight/obese: 9.510.9kg (6.4-5.4-13.415.0).

Conclusion

Optimal GWG ranges are lower for this ~~short stature migrant~~ population with short stature compared to existing international guidelines. Clinical and contextual factors must be considered when implementing GWG recommendations for this, and other marginalized and short-stature populations.

Introduction

Weight gain during pregnancy is widely recognized as an important determinant of maternal, delivery and neonatal outcomes[1–4]. Excessive gestational weight gain (GWG) has been linked to adverse outcomes such as gestational diabetes mellitus, hypertensive disorders of pregnancy, preterm birth, caesarean section and neonates born large for gestational age. Inadequate GWG has been associated with preterm birth~~,~~ and small for gestational age [3,4].

The National Academy of Medicine (NAM), formerly known as the Institute of Medicine (IOM) produced GWG recommendations in 1990, which were updated in 2009, and used data from women in the United States of America and Western Europe to define optimal GWG[5–7]. However, a 2016 review showed that the NAM recommendations were derived from a combination of studies, between which there was significant heterogeneity in methodology and statistical analysis[8].

To produce GWG guidelines across a diverse group of countries, data was used from the Intergrowth-21 (IG-21) prospective cohort [6]. This study incorporated 4,607 women and used strict inclusion and exclusion criteria to define a healthy pregnancy and neonatal outcome to create GWG guidelines for women with a normal BMI of 18.5-24.9 kg/m^2 (based on WHO BMI classification) and a height of 153cm or more[6,9].

However, a 2021 study by Lee et al., in a marginalised group of women living along the Thailand-Myanmar found that short maternal stature was associated with lower GWG and suggested that height should be considered with first-trimester BMI when assessing ideal GWG during pregnancy[5]. This population has an average height of 151.5cm, with almost 60% of women below the IG-21 cut-off for height of 153cm[5]. Lee et al (2021) reported that appropriate for gestational age (AGA) in term newborns was achieved by 80% of women even though only 25% achieved GWG within NAM recommendations[5].

There is therefore a gap in the GWG literature for ~~short-stature~~ populations of short-stature who are at risk of compromised nutrition before and during pregnancy[5]. ~~This study aimed to provide estimates for optimal GWG ranges, by Asia-Pacific Body Mass Index (BMI) group and height for the population living along the Thai-Myanmar border.~~ To inform the development of population-specific recommendations, this study investigated~~s~~ optimal GWG, by height and Body Mass Index (BMI), in a minority migrant and refugee population living along the Thailand-Myanmar border. The findings from our

studyresults may also be applicable to other short stature populations of short stature such as those from Nepal, East Timor and Bangladesh. globally, across socio-cultural contexts and healthcare services.

Methods

Ethical approval

Ethical approval was obtained through Oxford Tropical Research Ethics Committee (OxTREC reference 531-24) and the local body representing the community: Tak Province Community Advisory Board; T-CAB (TCAB202405). The T-CAB group is composed of community leaders who were consulted on the study design, process, and outcomes of interest[10].

Study setting

Shoklo Malaria Research Unit (SMRU), based in Mae Ramat, Thailand, is a field-based research organisation[11]. SMRU undertakes a combination of humanitarian clinical services and context-relevant research on both sides of Thailand-Myanmar border[12]. SMRU offers free antenatal, labour and delivery care to marginalised populations on the border[12]. SMRU served both refugee and migrant populations, however, healthcare services for refugee women were handed over to another humanitarian organisation in 2016. Thus, SMRU now supports Thailand's Public Health Department by filling a gap in care for migrant and displaced populations.

Study design

All pregnancy records from women with singleton pregnancies born at 28 weeks or later, between 01/01/2004 and 31/12/2023 were extracted from the SMRU electronic database. Antenatal and birth records are routinely collected and entered in a central electronic database which is securely managed by a local data team. The clinical information was gathered from medical documents that were originally maintained on paper and transitioned to electronic formats in late 2008. Records on

paper between 1986 and 2008 were digitized and incorporated into an electronic database to facilitate retrieval of antenatal care and birth outcome data. After 2008, all records have been entered directly into the electronic database.

The dataset for the purposes of this research was first accessed on 23/06/2024 and data extraction from the database was completed by 30/06/2024. When required for clarification, paper-based records were checked, which are stored at the SMRU offices. This was completed by 30/07/2024. All records were anonymised and identifiable information from the women was removed prior to analysis.

Participant inclusion/exclusion~~s~~ and study variables

A process of ~~further~~ data extraction based on inclusion and exclusion criteria was followed ~~(Fig 1)~~. Only records of singleton pregnancies with an estimated gestational age (EGA) of less than 14 weeks at first antenatal visit ~~were included~~, and at least two weight measurements over the course of the pregnancy were included. Women with last maternal weight recorded >4 weeks prior to delivery, pre-existing conditions such as hypertension, diabetes mellitus, HIV, moderate to severe anaemia at the first antenatal care visit and delivery prior to 28 weeks' gestation (spontaneous abortion) were excluded. Iatrogenic preterm births and neonates with major congenital abnormalities were also excluded. The small number of neonates with major congenital abnormalities were excluded due to their possible skewed birth weight distributions, such as being small or large for gestational age, which could confound the assessment of the relationship between maternal weight gain and neonatal outcomes. Additionally, certain congenital abnormalities can directly or indirectly influence GWG.

First trimester BMI (<14 weeks gestation) was used to represent pre-pregnancy BMI, as it has been shown to be an accurate approximation in several international cohorts[13–15]. The date of measurement of last maternal weight was used to calculate GWG and was required to be within four weeks of delivery to be included in the analysis. Trained midwives collected weight measurements at first ANC consultation and at each follow-up visit using mechanical Salter scales with 0.5kg precision. Gestational age was calculated by ultrasound, using crown-rump length between 9+0 to 13+6 weeks. The quality of scanning in this population has been confirmed and is reliable[16,17].

SGA, AGA and LGA classification was undertaken by calculating sex-, ethnicity- and gestational age-adjusted birthweight centiles using the Perinatal Institute's Gestation Related Optimal Weight (GROW) customised bulk centile calculator V.8.0[18]. GROW offers the advantage of region-specific classification and has coefficients to represent over 100

country-of-origin groups. Based on the average height, the Myanmar standards were used ~~F~~for women of Burmese ethnicity ~~women~~, ~~the Myanmar standards were used~~ and the Nepalese standards most closely matched ~~. For the Karen and other ethnicities, Nepalese standards as~~ the average height of Karen and other ethnicities in the dataset~~.~~ ~~most closely matches the average height of Nepalese women~~[19].

Neonates below the 10th percentile were classified as SGA, while those above the 90th percentile were classified as LGA[20,21]. Neonates at and between the 10th and 90th percentiles were classified as AGA.

Based on the literature and the availability of data in the cohort, the following maternal, ~~delivery~~ birth and neonatal outcomes were considered for further analysis in relation to GWG: gestational diabetes mellitus (GDM), hypertensive disorders of pregnancy (HDoP), caesarean section (CS), preterm birth (birth <37 weeks' gestation), small-for gestational age (SGA) and large-for ~~gestataional~~gestational age (LGA). HDoP included gestational hypertension, pre-eclampsia and eclampsia.

Statistical analysis

All statistical analyses were conducted using R (Version 4.2.1) and RStudio (Version 2024.04.0+735). Initial descriptive statistics were performed according to Asia-Pacific BMI categories which were defined as: underweight (<18.5 kg/m²), normal weight (18.5–22.9 kg/m²), overweight (23.0–24.9 kg/m²), and obese (≥25.0 kg/m²)[22,23]. For the purposes of this analysis, overweight and obese women were categorized into a single group as the narrow overweight range limited its utility as a distinct category and separating the overweight and obese groups resulted in small, underpowered groups.

Continuous variables with a normal distribution were summarised using mean ± standard deviation (SD), otherwise median and interquartile range (IQR; 25th-75th percentiles) were reported. Categorical variables were expressed as frequencies ~~and~~ with percentages (n(%)). The chi-squared test and Cochran-Armitage test for trend were used to assess associations between categorical variables across BMI groups. Statistical significance was set at $p < 0.05$.

Univariable and multivariable logistic regression models were used to assess the relationships between GWG and outcomes. Multivariable models were adjusted for known confounders including age, parity, smoking status and ethnicity, based on the literature[3,24]. To address potential collinearity between age and parity, a categorisation to combine age and parity into six groups was created, and was used as a single categorical variable in the models. The groups were defined as ~~follows~~ age <20 and~~&~~ nulliparous~~ity=0~~; age <20 and parity ≥1; age 20-29 and~~&~~ nullipar~~ity=0~~ous; age 20-29 ~~&~~and parity ≥1; age ≥ 30 ~~&~~and nullipar~~ity = 0~~ous; age ≥30 and~~&~~ parity ≥1.

Formatted: Font: Times New Roman, Font color: Text 1

Formatted: Font: Times New Roman, Font color: Text 1

Adjustment was also made for year (2004-2012, 2013-2016, 2017-2020, 2021-20234). This accounted for method of GDM screening, as this changed from no screening before 2013, to risk-factor based screening in 2013-2020, to full population screening after 2020[25] and also for the change in care provision to migrant populations only from 2017. Additionally, adjustment was made for malaria (treated during pregnancy), as it is a common infection in this area, and is associated with adverse outcomes including HDoP, SGA, preterm birth and stillbirth[16,26,27]. Although literacy and socioeconomic statusSES are also possible confounders[4,24], reliable measures of these variables were not available within the dataset.

As visual inspection of the association between GWG and adverse outcomes indicated nonlinear relationships, natural cubic splines with logistic regression models were used. Natural cubic splines were chosen to mitigate overfitting, especially at the boundaries of the data.To allow for potential nonlinear relationships between GWG and adverse outcomes, logistic regression models were fitted incorporating natural (restricted) cubic splines for GWG. Natural cubic splines were used to mitigate overfitting, especially at the boundaries of the data[28]. The splines were constructed as a series of polynomial functions, each defined within a specific interval of GWG. Each spline was joined smoothly using knots, ensuring that the overall function was continuous. The optimal number of knots was determined though visualisation of the relationship between predictor and outcome variables. All models were constructed using 2 to 3 degrees of freedom (i.e. 3 to 4 knots) to maintain simplicity while capturing essential patterns.

Formatted: Normal (Web)

Using these models, predicted probabilities were calculated for each adverse outcome across the range of GWG. For each weight, the predicted probabilities of all adverse outcomes were summed to derive a composite risk score. The minimum composite risk score was used to define the optimal GWG, with the optimal range corresponding to a composite score within 5% of the minimum risk. This methodology uses elements similar to those used by Choi et al. (2017) for a cohort of Korean women, by Morisaki et al. (2017) who considered GWG in Japanese women, and by Ee et al., in a Singaporean cohort (2014) [29–31].

Results

Figure 1 illustrates the selection process for the study cohort. From a total of 44,364 women with singleton pregnancies and deliveries at ≥28 weeks' gestation between January 1, 2004, and December 31, 2023, 25,565 women (58.0%) were excluded due to enrolment at ≥14 weeks' gestation (n=24,016) or incomplete height and weight data (n=1,549). Additionally, women with last maternal weight recorded >4 weeks prior to delivery (n=1,262), pre-existing conditions such as hypertension, diabetes

mellitus, or HIV (n=75), moderate to severe anaemia at the first antenatal care visit (HCT <25%, n=48), iatrogenic preterm births (N=174) and neonates with major congenital abnormalities (n=46) were excluded.

The final cohort analysed consisted of 17,194368 women., with outcome data available for 15,927 neonates. The remaining neonates were delivered either at home, in Myanmar, or in hospitals not affiliated with SMRU clinics.

Fig Figure **1.** Flow of study participants.

Maternal demographic characteristics

Table 1 shows the characteristics, by Asia-Pacific BMI group, of the 17,194368 women who met the inclusion criteria for the study between 2004 and 2023. More than half of women, 10,115213 (58.8%) had a normal first trimester BMI, while 3,01938 (17.65%) were underweight (minimum BMI 13.5 kg/m^2), and 4,060117 (23.67%) overweight or obese by Asia-Pacific BMI standards (Table 1). There were 2089 women with an overweight BMI (23 - 24.9kg/m²) and 1971 women with an obese (≥ 25kg/m²) first trimester BMI (S1 Table). For context, the proportion of women in the cohort with BMI ≥30 kg/m² (obese by WHO International Standards) was only 2% (363 of 17,368 women), with a maximum BMI of 43kg/m^2.

In the underweight BMI group, the range was 4.9 kg/m² (13.5 to 18.4 kg/m²) and the range for the normal BMI category was bound at 4.4 kg/m² (18.5-22.9 kg/m²). In the overweight BMI group, the range was 1.9 kg/m² (23 - 24.9kg/m²), while for the obese group it was much wider, 18 kg/m² (25-43kg/m^2). The proportion of women in the cohort with BMI ≥30 kg/m² (obese by WHO International Standards) was only 2% (356 of 17,194 women). The distribution of BMI can be seen in S1 Fig (supplementary).

The mean height (SD) for the cohort was 151.4cm (5.4cm), and more than half of women (10,205318, 59.4%) had a height less than 153cm. The distribution of height in the cohort can be seen in the supporting information document (S2 Fig S1). Mean GWG for the full cohort was 9.3kg (SD 4.0kg), which decreased with increasing BMI category (test for trend p<0.001). Most women with a BMI <18.5kg/m^2 gained below NAM recommendations (2,2662281, 75.1%); only a quarter (696700, 23.1%) gained within NAM guidelines. A similar pattern was present in the women with normal BMI, where 7,30588 (72.23%) gained below NAM guidelines, while 2,36375 (23.43%) gained within NAM recommendations, and 44750 (4.4%) gained above NAM recommendations. In the overweight and obese group almost half (1,755571, 43.2%) of women gained within NAM recommendations, with 1,15482 (28.47%) gaining below and 1,15164 (28.3%) gaining above NAM.

Most women had a vaginal birth (16,337422 (954.06%)) while 857945 (5.04%) delivered by caesarean section. Rates of caesarean section increased with increasing BMI category from 2.83.3% (underweight group) to 9.07% (overweight/-obese group) (test for trend p<0.001).

Table 1. Maternal demographic and outcome descriptive statistics by Asia-Pacific BMI categories.

Formatted: Font: Bold

Variable	All Women, n (%)	Underweight (<18.5 kg/m²)	Normal (18.5–22.9 kg/m²)	Overweight & Obese (≥23 kg/m²)
**Total Women**	17,194368	3,01938 (17.65)	10,115213 (58.8)	4,060117 (23.77)
**Median Age (IQR), years**	25 (20–30)	23 (20–28)	24 (20–30)	28 (23–33)
**Nulliparous (%)**	6231 (36.2)	1345 (44.6)	3933 (38.9)	953 (23.5)
**Primigravid (%)**	6291 (36.2%)	1352 (44.5%)	3972 (38.9%)	967 (23.5%)
**Parity*Age groups[b]**				
Age <20 & Parity 0				
Age <20 & Parity ≥1				
Age 20-29 & Parity 0				
Age 20-29 & Parity ≥1				
Age ≥30 & Parity 0				
Age ≥30 & Parity ≥1				
**Ethnicity**				
Karen (%)	8,9949,093 (52.34)	1,38396 (45.86.0)	5,42780 (53.7)	2,184217 (53.8)
Burmese (%)	4,160200 (24.2)	8847 (29.32)	2,15478 (21.3)	1,12235 (27.6)
Other (%)	4,04075 (23.5)	7525 (24.9)	2,53455 (25.10)	75465 (18.6)
**Mean Height (SD), cm**	151.4 (5.4)	152 (5.5)	151.1 (5.4)	151.8 (5.4)
**Height <153 cm (%)**	10,205318 (59.4)	1,692706 (56.02)	6,198262 (61.3)	2,31550 (57.01)
**Mean GWG (SD), kg**	9.3 (4.0)	10.2 (3.5)	9.5 (3.8)	8.21 (4.4)
**NAM[a] GWG Recommendation (kg)**				
Below NAM[a] (%)	10,725851 (62.45)	2,266281 (75.1)	7,30588 (72.23)	1,15482 (28.47)
Within NAM (%)	4,81446 (28.07.9)	696 700 (23.10)	2,36375 (23.43)	1,75571 (43.20)
Above NAM (%)	1,65571 (9.6)	57 (1.9)	44750 (4.4)	1,15164 (28.3)

**Smokers (%)[b]**	2,65070 (15.4)	585683 (18.5)	1,67383 (16.5)	41924 (10.3)
**Hypertensive Disorder (%)**	1,12764 (6.67)	1105 (3.68)	55780 (5.57)	4609 (11.34)
**Gestational Diabetes Mellitus (%)[c]**	62837 (3.7)	77 (2.65)	21920 (2.2)	33240 (8.23)
**Malaria in Pregnancy (%)**	1,70316 (9.9)	3859 (12.8)	1,07785 (10.6)	2412 (511.95)
**Caesarean Section (%)**	857945 (5.04)	8699 (2.83.3)	40547 (4.04)	36699 (9.07)

[a]NAM = National Academy of Medicine. [b]Gestational Diabetes Mellitus, nN=7 values missing. [c]Smoking, nN=47 values missing. ; [b] As used in the adjusted analysis

Delivery and neonatal outcomes

Birth outcome was available for all 17,194363 neonates (FFig 1), of which 17,109242 (99.53%) were live births (Ttable 2). Of all neonates, 1,048200 (6.19%) were born preterm. Birthweight for gestational age centiles were calculated for 15,783926 (91.87%) neonates who had a birthweight measured within 72 hours of delivery. While most babies were born AGA (12,402373 (787.67%)), nearly one in six five were SGA (2,463 722 (157.61%)), and a minority, one in twenty, (918824 (5.82%)) were LGA.

Table 2. Neonatal descriptive statistics by maternal Asia-Pacific BMI categories.

**Variable**	**All**	**Underweight (<18.5 kg/m²)**	**Normal (18.5–22.9 kg/m²)**	**Overweight & Obese (≥23 kg/m²)**
**All births with known outcome (%)**	17,194363	3,01937 (17.65)	10,115211 (58.8)	4,060115 (23.67)
Live births	17,109242 (99.53)	2,9993,016 (99.33)	10,065137 (99.53)	4,04589 (99.64)
Stillbirths	85121 (0.57)	2021 (0.77)	5074 (0.57)	1526 (0.46)
Male[a]	8,648740 (50.3)	1,494504 (49.5)	5,096149 (50.4)	2,05887 (50.7)
Median EGA (IQR), weeks	39.3 (38.4–40.1)	39.2 (38.3–40.0)	39.3 (38.4–40.1)	39.4 (38.5–40.2)
Preterm births <37 weeks (%)	1,048200 (6.19)	25970 (8.69)	645730 (67.41)	144200 (34.59)
**Birthweight measured <72 hours[b]**	15,783926	2,67288 (16.9)	9,284369 (58.8)	3,82769 (24.23)
Mean Birthweight (SD), g	2,98273.08 (44657.86)	2,84339.51 (4405.92)	2,9590.03 (43446.48)	3,13424.65 (43852.31)
SGA (%)	2,463722 (157.61)	470518 (179.63)	1,414560 (156.27)	579644 (156.16)
AGA (%)	12,402373 (787.67)	2,06143 (776.10)	7,3335 (798.03)	32,008995 (787.64)

Formatted: Superscript

Formatted: Font: Bold

Formatted: Superscript

Formatted: Superscript

Formatted: Superscript

LGA (%)	918824 (5.82)	14124 (54.36)	537471 (5.80)	24029 (6.35.9)

[a]Male baby, nN=6 values missing.

[b]Birthweight for gestational age centiles were calculated for 15,783 (91.8%) neonates who had a birthweight measured within 72 hours of delivery

Calculation of optimal GWG

Two thirds of the pregnancies did not experience HDoP, GDM, CS, spontaneous preterm birth, SGA or LGA (11,309 women (65.8%)), 4,878 (28.4%) had one adverse outcome, and less than 6% experienced two or more adverse outcomes (1,007 women (5.6%)). The six outcomes of HDoP, GDM, CS, spontaneous preterm birth, SGA and LGA were plotted as predicted probabilities against GWG using smoothed restricted cubic splines for the BMI categories of underweight, normal and overweight/obese, each for two height categories of <153 and ≥ 153cm (Figsg. 2-4). The GWG at which the composite risk score for adverse outcomes was lowest was used to calculate an optimal GWG, and range (Table 3).

In the cohort of women with an underweight BMI in the first trimester and height <153cm, the optimal GWG was 12.11 (10.0-14.59.9-14.5kg), while in the underweight group with height of 153cm or more, the optimal GWG was higher, at 13.15kg (11.05-15.14.5kg) (Fig 2). A similar pattern was seen across both the normal BMI and overweight/obese groups, in which the women with a height <153cm had a lower optimal GWG than the taller women (Figs 3 & 4). In the women with a normal first trimester BMI, optimal GWG in the <153cm cohort was 10.4kg (8.0-12.9kg), while for normal BMI women with height ≥153cm, optimal GWG was 12.33kg (9.710.3-15.36kg) (Fig 3). In the combined overweight/obese first-trimester BMI group, in the women with height <153cm, optimal GWG was 56.39kg (3.17-8.59.7kg), while in those ≥153cm, optimal GWG was 9.510.9kg (6.45.4-13.45.0kg) (Fig 4). Figures S32, S43 and S5 Figs4 in the supporting information show the calculated optimal GWG ranges superimposed on the on the predicted probabilities of adverse maternal and neonatal outcomes, stratified by BMI and height categories.

Figure 2. Predicted probabilities with 95% confidence intervals (y-axis) of adverse outcomes across GWG (x-axis) for women with an underweight first trimester BMI. Calculated using logistic regression models incorporating cubic splines. NAM GWG recommendations superimposed as vertical grey lines at 12.5 and 18.0kg (a) Women with underweight BMI and height <153cm, N=1,692706; (b) Women with underweight BMI and height ≥153cm, N= 1,32732.
Key: CS=Caesarean Section; GDM=Gestational Diabetes Mellitus; HDoP=Hypertensive Disorders of Pregnancy; LGA=Large for Gestational Age; Preterm=Preterm birth; SGA=Small for Gestational Age.

Figure 3. Predicted probabilities with 95% confidence intervals (y) of adverse outcomes across GWG (x) for women with a normal first trimester BMI. Calculated using logistic regression models incorporating cubic splines. NAM GWG

Formatted: Font: 10 pt, Font color: Text 1, English (United Kingdom)

Formatted: MDPI_2.2_heading2, Indent: First line: 0.49", Space After: 0 pt

Formatted: Font: 10 pt

Formatted: Font: 10 pt, Bold

Formatted: Font: 10 pt

Formatted: Font: 10 pt, Bold

Formatted: Font: 10 pt

Formatted: Font: 10 pt, Bold

Formatted: Font: 10 pt

Formatted: Font: 9 pt

Formatted: Font: 10 pt

Formatted: Font: 10 pt, Bold

recommendations superimposed as vertical grey lines at 11.5 and 16.0kg (a) Women with normal BMI and height <153cm, N=6,198~~262~~; (b) Women with normal BMI and height ≥153cm, N=3,917~~51~~.

Figure 4. Predicted probabilities with 95% confidence intervals (y) of adverse outcomes across GWG (x) for women with an overweight or obese first trimester BMI. Calculated using logistic regression models incorporating cubic splines. NAM GWG recommendations superimposed as vertical grey lines at 7.0 and 11.5kg (a) Women with overweight or obese BMI and height <153cm, N=2,315~~50~~; (b) Women with overweight or obese BMI and height ≥153cm, N=1,745~~67~~.

Table 3. Optimal GWG ranges based on minimisation of composite risk of adverse outcomes per BMI category.

**Asia-Pacific BMI category (kg/m²)**	**Optimal GWG (range), kg full cohort**	**Optimal GWG (range), kg height <153cm**	**Optimal GWG (range), kg height ≥153cm**	**NAM GWG recommendation, kg[a]**
Underweight (<18.5)	12.4<br>(10.2– 14.8)<br>N=3,019	12.1<br>(10.0-14.5)<br>N=1,692	13.1<br>(11.0-15.1)<br>N=1,327	12.5-18.0
Normal (18.5-22.9)	11.0<br>(8.3 – 14.0)<br>N=10,115	10.4<br>(8.0-12.9)<br>N=6,198	12.3<br>(9.7-15.3)<br>N=3,917	11.5-16.0
Overweight/Obese (≥23)	9.0<br>(5.6-12.1)<br>N=4,060	5.3<br>(3.1-8.5)<br>N=2,315	9.5<br>(6.4-13.4)<br>N=1,745	Overweight: 7-11.5<br>Obese: 5.0-9.0

~~**Table 3.** Optimal GWG ranges based on minimisation of composite risk of adverse outcomes per BMI category.~~

~~**Asia-Pacific BMI**~~ **Asia-Pacific BMI category (kg/m²)**<br>Underweight (<18.5)<br>Normal (18.5-22.9)<br>Overweight/Obese (≥23)<br>~~**category (kg/m²)**~~	~~**Optimal GWG (range), kg full cohort**~~	~~**Optimal GWG (range), kg height <153cm**~~	~~**Optimal GWG (range), kg height ≥153cm**~~	~~**NAM GWG recommendation, kg[a]**~~
~~Underweight (<18.5)~~	~~12.6<br>(10.5 – 15.4)<br>N=3,038~~	~~12.1<br>(9.9-14.5)<br>N=1,706~~	~~13.5<br>(11.5-15.4)<br>N=1,332~~	~~12.5-18.0~~

Formatted: Font: 10 pt
Formatted: Font: 10 pt, Bold
Formatted: Font: 10 pt
Formatted: Font: 10 pt, Bold
Formatted: Font: 10 pt
Formatted: Font: 10 pt, Bold
Formatted: Font: 10 pt
Formatted: Font: 10 pt, Bold
Formatted: Font: 10 pt
Formatted: Font: 10 pt, Bold
Formatted: Font: (Default) Times New Roman, 10 pt
Formatted: Font: (Default) Times New Roman, 10 pt, Bold
Formatted Table
Formatted: Space After: 0 pt
Formatted: Space After: 0 pt
Formatted: Space After: 0 pt
Formatted: Space Before: 0 pt, After: 0 pt

~~Normal (18.5-22.9)~~	~~11.3~~<br><br>~~(8.6 – 14.0)~~<br>~~N=10,213~~	~~8.9~~<br><br>~~(7.1-11.3)~~<br>~~N=6,262~~	~~13.0~~<br><br>~~(10.3-16.0)~~<br>~~N=3,951~~	~~11.5-16.0~~
~~Overweight/Obese (≥23)~~	~~7.7~~<br>~~(4.2-11.4)~~<br>~~N=4,117~~	~~6.9~~<br>~~(3.7-9.7)~~<br>~~N=2,350~~	~~10.9~~<br>~~(5.4-15.0)~~<br>~~N=1,767~~	~~Overweight: 7-11.50~~<br>~~Obese: 5.0-9.0~~

[a]For reference: NAM GWG recommendations are based on WHO BMI classifications which are defined as follows: underweight (<18.5 kg/m^2), normal (18.5–24.9 kg/m^2), overweight (25.0–29.9 kg/m^2), obese (≥30.0 kg/m^2).

Discussion

GWG ranges based on first-trimester Asia-Pacific BMI categories were calculated to minimize the risks of adverse pregnancy and birth outcomes. In general, the GWG ranges for the migrant and refugee population were lower than the NAM guidelines. For women with height less than 153cm and normal BMI, the optimal GWG was 10.4kg with range 8.0-12.9 kg compared with 11.5-16.0 from the NAM guidelines. ~~In general, the GWG ranges for the migrant, short stature population were lower than the NAM guidelines; with both the underweight and overweight/obese groups although over 3000, still limited~~ .

The NAM guidelines, primarily derived from women in North America (average height of 162 cm) and Western Europe (166 cm), recommend a GWG of 11.5-16kg at term in normal BMI women by WHO BMI standards[7,19]. IG-21, which included only women with a height of 153cm or more, provide a 50th centile GWG recommendation of 13.2kg (10th-90th centiles: 8.3-19.4kg) for normal BMI women (by WHO BMI standards) at 39 weeks' gestation (equivalent to the mean gestation at delivery in the migrant and refugee cohort)[6]. In this analysis, when restricted to women of 153cm or more, optimal GWG (12.3~~3~~kg, range: 9.7~~10.3~~-15.3~~16~~) was comparable to~~-~~ the ~~both the NAM and~~ IG-21 range~~s~~. However, for women shorter than 153cm, the optimal GWG was lower (10.4~~8.9~~kg, range: 8.0-12.9~~7.1-11.3~~). For the underweight BMI category, in which NAM recommends a GWG of 12.5-18kg, in this analysis optimal GWG was 13.1~~5~~kg (11.0~~5~~-15.1~~4~~) in women ≥153cm, while in underweight women <153cm, optimal GWG was lower: 12.1~~1~~kg (~~9.9-14.5~~10.0-14.5).

Morisaki et al. (2017)~~,~~ included 104 070 Japanese women with singleton pregnancies to find the GWG with lowest risk of adverse outcomes (SGA, preterm birth, pre-eclampsia and complicated delivery: CS, forceps or vacuum delivery, obstructed labour, PPH)[30]. Morisaki et al. ~~(2017)~~ calculated the optimal weight gain to minimise adverse outcomes, by BMI groups (kg/m^2): BMI 17.0–18.4: 12.2 kg; BMI 18.5–19.9: 10.9kg; BMI 20–22.9: 9.9kg; 23–24.9: 7.7kg; and 25–27.4:

4.3kg[30]. This Japanese population had a mean height of 158cm (lower than the average for American and European populations) and the optimal GWG ranges were close to those developed for the ~~short stature migrant~~ cohort of women with short stature anaylsed here[30].

An additional finding in the current analysis of women living along the Thailand-Myanmar border is that the proportion of women with BMI ≥30 kg/m² is only 2%, which contrasts with countries such as the US, where this proportion is estimated to be higher than 20%[32,33]. In the Thai-Myanmar border population, where only a small proportion of women fall into the overweight/obese category, the overall GWG distribution may be skewed by the higher proportion of women at the lower end of the BMI spectrum. ~~-~~This finding highlights the role that BMI distribution plays in shaping GWG patterns within a population and may also explain why the ~~wide~~ interval for GWG in the women with first trimester overweight and obese BMI was wider than that calculated for normal and underweight BMI groups (Table 3). This wider interval can be attributed to the ~~extensive~~ wide variation, ~~range~~ of first trimester BMI ~~covered~~ in the overweight and obese group, ~~spanning~~ which ranged from 23 to 43kg/m². In the underweight BMI group, the range was only 4.9 kg/m² (13.5 to 18.4 kg/m²) and the range for the normal BMI category was bound at 4.4 kg/m² (18.5-22.9 kg/m²).~~Such a broad range indicates that the variations in weight gain are considerably more pronounced compared to the underweight (BMI range of 13.5 to 18.4 kg/m²) and normal BMI (18.5-22.9 kg/m²) categories. .~~ Thus, a~~A~~pplying guidelines developed in populations with different BMI distributions may not fully capture the nuanced needs and~~of the~~ unique demographic and health characteristics of all populations.

This analysis also ha~~ds~~ other limitations. While in this study, the optimal GWG and ranges have been calculated, the pattern and timing of weight gain across gestation is equally, if not more, important[34]. Longitudinal data is crucial for understanding the impact of GWG during different trimesters, and to account for changes in maternal and foetal development across various stages of pregnancy[34]. Furthermore, data on literacy and socioeconomic status, which can influence health and well-being of pregnant women and affect the GWG, was limited for this cohort~~ was limited~~, thus could not be adjusted for in ~~this study~~the analysis. ~~All~~However, we do know that all ~~women were~~women in this cohort were seeking services in a humanitarian healthcare setting~~s. suggesting a low and undifferentiated socioeconomic status.~~

We were unable to adjust for within subject correlation due to repeat pregnancies (i.e., mothers being included in the dataset more than once). Data collection for this was only available for more recent pregnancies and as data collection continues, this is an aspect of analysis that will be included in future studies.

Formatted: Font: 10 pt, Font color: Text 1

Formatted: Line spacing: 1.5 lines

An additional limitation to the approach used for calculating optimal GWG in this analysis is that the composite scores are likely overestimating risk because the interrelated nature of certain adverse outcomes is not accounted for. For instance, GDM, CS and LGA may~~often~~ co-occur and the calculation of the composite score which considers each of these as separate risks might overestimate the total calculated risk. However, in this cohort the proportion of women with two or more adverse outcomes was less than 6%, thus, the impact of "overestimating" risk was likely minor. Instead, the GWG ranges reported here would be most strongly influenced by the two thirds of women who had no adverse events.

Additionally, the screening and diagnosis of GDM has changed over time in this population, and thus the estimation of risk for GDM may not be representative of the true rates in this population. Future analyses should consider an approach to account for the potential overlap between adverse outcomes to provide a more accurate risk assessment.

To get an accurate reflection of GWG patterns and classify women into BMI groups, the data in this analysis was limited to women who had their first antenatal care (ANC) visit before 14 weeks' gestation. This process excluded approximately 40% of women who gave birth between 2004 and 2023, reflective of the real-world situation where many women do not access care early in gestation or do not have a documented first trimester BMI. If, for example, a woman at 20 weeks' estimated gestational age presents for a first time ANC visit, already having gained weight, it would be unclear which recommendations should be followed. Additionally, classifying BMI, a continuous variable, into groups comes at the expense of classifying women with a BMI of 22.8 and 23.0 as more different than similar, and then applying differing GWG recommendations to these women. Likewise, the height categories applied in this analysis were broad and may lead to recommendations that do not apply well to rare outliers (<140 cm or >160 cm) in this population.

A third challenge of BMI categorisation wa~~i~~s ~~that~~ the sample sizes per BMI group~~ in this analysis were vastly different~~. The relatively smaller number of women with overweight (N=2089) and obese (N=1971) first-trimester BMIs meant that when the analysis was run for these groups individually the results were underpowered.~~,~~ ~~h~~Hence overweight and obese were combined ~~into a single group~~ for the analysis although overweight and obese women are not a homogenous group and ideally these groups would be analysed separately.

To address the limitations of BMI categorisation, further research should focus on the development of an interactive tool into which height, current weight and gestation can be entered, with an output of optimal GWG based on these criteria. Alternatively, methods of estimating GWG which do not rely on first trimester or pre-pregnancy BMI could also be considered. One example of this is mid-upper arm circumference (MUAC) measurement, which was proven to be strongly associated with first-trimester BMI and GWG in Darling et al.'s 2023 systematic review and meta-analysis [24]. Future studies could consider

Formatted: Space After: 0 pt, Line spacing: Double

Formatted: Font: 10 pt

Formatted: Font color: Text 1

Formatted: Font color: Text 1

Formatted: Font color: Text 1

Formatted: Font color: Text 1

Formatted: Font color: Text 1

Formatted: Font color: Text 1

Formatted: Line spacing: Double

how MUAC changes over gestation and if MUAC can be used to accurately represent BMI or GWG patterns, especially in women who do not enter antenatal care in first trimester.

Conclusion

This study contributes to the growing body of literature on GWG by focusing on a unique and marginalized population of ~~short-stature~~ women of short-stature living along the Thailand-Myanmar border. The international NAM guidelines, based on taller populations from North America and Western Europe, overestimate the optimal GWG for this migrant and refugee cohort, which has an average height of 151.4cm. The GWG ranges developed in this study can be applied in clinical practice when advising women attending antenatal care at SMRU on the amount of weight to gain during pregnancy. There are limited data internationally on pregnant women with short stature globally, thus the findings from our study~~These~~ may also have applicability to other ~~short stature~~ populations of short stature worldwide. ~~.~~

Supporting information

S1 File. Figure S1-S5~~4~~. Table S1-3~~1~~.

(PDF~~DOCX~~)

Formatted: Indent: First line: 0"

Acknowledgments

We recognize the efforts of the maternal and child health team at SMRU whose commitment to patient care and data collection has been foundational to this research and represents the collective work of many people over many years.

Author contributions

Conceptualization: Mary Gouws, Sue J. Lee, Wirichada Pan-ngum, Rose McGready.

Methodology: Mary Gouws, Sue J. Lee.

Data curation: Aung Myat Min, Nay Win Tun, Mary Ellen Gilder, Taco Jan Prins, Widi Yotyingaphiram, Mupawjay Pimanpanarak, Jacher Viladpai-Nguen, Nuttapol Panachuenwongsakul, François H Nosten, Rose McGready.

Formal analysis: Mary Gouws.

Supervision: Sue J. Lee, Wirichada Pan-ngum, Rose McGready.

Writing – original draft: Mary Gouws.

Writing – review & editing: Sue J. Lee, Wirichada Pan-ngum, Aung Myat Min, Nay Win Tun, Mary Ellen Gilder, Taco Jan Prins, Widi Yotyingaphiram, Mupawjay Pimanpanarak, Jacher Viladpai-Nguen, Nuttapol Panachuenwongsakul, François H Nosten, Rose McGready.

References

1. Crane JMG, White J, Murphy P, Burrage L, Hutchens D. The Effect of Gestational Weight Gain by Body Mass Index on Maternal and Neonatal Outcomes. Journal of Obstetrics and Gynaecology Canada. 2009;31: 28–35. doi:10.1016/S1701-2163(16)34050-6

2. LifeCycle Project-Maternal Obesity and Childhood Outcomes Study Group. Association of Gestational Weight Gain With Adverse Maternal and Infant Outcomes. JAMA. 2019;321: 1702–1715. doi:10.1001/jama.2019.3820

3. Goldstein RF, Abell SK, Ranasinha S, Misso M, Boyle JA, Black MH, et al. Association of Gestational Weight Gain With Maternal and Infant Outcomes: A Systematic Review and Meta-analysis. JAMA. 2017;317: 2207–2225. doi:10.1001/jama.2017.3635

4. Goldstein RF, Abell SK, Ranasinha S, Misso ML, Boyle JA, Harrison CL, et al. Gestational weight gain across continents and ethnicity: systematic review and meta-analysis of maternal and infant outcomes in more than one million women. BMC Med. 2018;16: 153. doi:10.1186/s12916-018-1128-1

5. Lee SJ, Hashmi AH, Min AM, Gilder ME, Tun NW, Wah LL, et al. Short maternal stature and gestational weight gain among refugee and migrant women birthing appropriate for gestational age term newborns: a retrospective cohort on the Myanmar-Thailand border, 2004-2016. BMJ Glob Health. 2021;6: e004325. doi:10.1136/bmjgh-2020-004325

6. Cheikh Ismail L, Bishop DC, Pang R, Ohuma EO, Kac G, Abrams B, et al. Gestational weight gain standards based on women enrolled in the Fetal Growth Longitudinal Study of the INTERGROWTH-21st Project: a prospective longitudinal cohort study. BMJ. 2016;352: i555. doi:10.1136/bmj.i555

7. Institute of Medicine (US) and National Research Council (US) Committee to Reexamine IOM Pregnancy Weight Guidelines. Weight Gain During Pregnancy: Reexamining the Guidelines. Rasmussen KM, Yaktine AL, editors. Washington (DC): National Academies Press (US); 2009. Available: http://www.ncbi.nlm.nih.gov/books/NBK32813/

8. Ohadike CO, Cheikh-Ismail L, Ohuma EO, Giuliani F, Bishop D, Kac G, et al. Systematic Review of the Methodological Quality of Studies Aimed at Creating Gestational Weight Gain Charts. Advances in Nutrition. 2016;7: 313–322. doi:10.3945/an.115.010413

9. Gestational Weight Gain | Intergrowth. [cited 3 Aug 2024]. Available: https://intergrowth21.com/tools-resources/gestational-weight-gain

10. Lwin KM, Peto TJ, White NJ, Day NPJ, Nosten F, Parker M, et al. The practicality and sustainability of a community advisory board at a large medical research unit on the Thai-Myanmar border. Health (Irvine Calif). 2013;5: 229–236. doi:10.4236/health.2013.52031

11. SMRU Clinics | Shoklo Malaria Research Unit. [cited 8 May 2024]. Available: https://www.shoklo-unit.com/humanitarian-activities/smru-clinics

12. The overlap between miscarriage and extreme preterm birth in a limited-resource setting on the Thailand-Myanmar border: a population cohort study - PMC. [cited 17 May 2024]. Available: https://www.ncbi.nlm.nih.gov/pmc/articles/PMC6305214/

13. Rangel Bousquet Carrilho T, M. Rasmussen K, Rodrigues Farias D, Freitas Costa NC, Araújo Batalha M, E. Reichenheim M, et al. Agreement between self-reported pre-pregnancy weight and measured first-trimester weight in Brazilian women. BMC Pregnancy Childbirth. 2020;20: 734. doi:10.1186/s12884-020-03354-4

14. Inskip H, Crozier S, Baird J, Hammond J, Robinson S, Cooper C, et al. Measured weight in early pregnancy is a valid method for estimating pre-pregnancy weight. Journal of Developmental Origins of Health and Disease. 2021;12: 561–569. doi:10.1017/S2040174420000926

15. Natamba BK, Sanchez SE, Gelaye B, Williams MA. Concordance between self-reported pre-pregnancy body mass index (BMI) and BMI measured at the first prenatal study contact. BMC Pregnancy Childbirth. 2016;16: 187. doi:10.1186/s12884-016-0983-z

16. Rijken MJ, Mulder EJH, Papageorghiou AT, Thiptharakun S, Wah N, Paw TK, et al. Quality of ultrasound biometry obtained by local health workers in a refugee camp on the Thai-Burmese border. Ultrasound Obstet Gynecol. 2012;40: 151–157. doi:10.1002/uog.11091

17. Rijken MJ, Lee SJ, Boel ME, Papageorghiou AT, Visser GHA, Dwell SLM, et al. Obstetric ultrasound scanning by local health workers in a refugee camp on the Thai–Burmese border. Ultrasound in Obstetrics & Gynecology. 2009;34: 395–403. doi:10.1002/uog.7350

18. Gestation Network, Perinatal Institute. Gestation Related Optimal Weight: Global Bulk Centile Calculator. [cited 18 Jun 2024]. Available: https://www.gestation.net/cc/about.htm

19. Average height for men and women worldwide. In: Worlddata.info [Internet]. [cited 13 May 2024]. Available: https://www.worlddata.info/average-bodyheight.php

20. Battaglia FC, Lubchenco LO. A practical classification of newborn infants by weight and gestational age. J Pediatr. 1967;71: 159–163. doi:10.1016/s0022-3476(67)80066-0

21. Chew LC, Verma RP. Fetal Growth Restriction. StatPearls. Treasure Island (FL): StatPearls Publishing; 2024. Available: http://www.ncbi.nlm.nih.gov/books/NBK562268/

22. World Health Orgnaisation, International Diabetes Institute. The Asia-Pacific Perspective: Redefining Obesity and its Treatment. World Health Organisation; 2020 Feb. Available: https://iris.who.int/bitstream/handle/10665/206936/0957708211_eng.pdf

23. Tham KW, Abdul Ghani R, Cua SC, Deerochanawong C, Fojas M, Hocking S, et al. Obesity in South and Southeast Asia—A new consensus on care and management. Obesity Reviews. 2023;24: e13520. doi:10.1111/obr.13520

24. Darling AM, Wang D, Perumal N, Liu E, Wang M, Ahmed T, et al. Risk factors for inadequate and excessive gestational weight gain in 25 low- and middle-income countries: An individual-level participant meta-analysis. PLOS Medicine. 2023;20: e1004236. doi:10.1371/journal.pmed.1004236

25. Prüst JT, Brummaier T, Wah M, Yee HH, Win NN, Pimanpanarak M, et al. Risk factor-based screening compared to universal screening for gestational diabetes mellitus in marginalized Burman and Karen populations on the Thailand-Myanmar border: An observational cohort. Wellcome Open Res. 2022;7: 132. doi:10.12688/wellcomeopenres.17743.2

26. Moore KA, Simpson JA, Scoullar MJL, McGready R, Fowkes FJI. Quantification of the association between malaria in pregnancy and stillbirth: a systematic review and meta-analysis. The Lancet Global Health. 2017;5: e1101–e1112. doi:10.1016/S2214-109X(17)30340-6

27. Harrington WE, Moore KA, Min AM, Gilder ME, Tun NW, Paw MK, et al. Falciparum but not vivax malaria increases the risk of hypertensive disorders of pregnancy in women followed prospectively from the first trimester. BMC Med. 2021;19: 98. doi:10.1186/s12916-021-01960-3

28. Gauthier J, Wu QV, Gooley TA. Cubic splines to model relationships between continuous variables and outcomes: a guide for clinicians. Bone Marrow Transplant. 2020;55: 675–680. doi:10.1038/s41409-019-0679-x

29. Choi SK, Lee G, Kim YH, Park IY, Ko HS, Shin JC. Determining optimal gestational weight gain in the Korean population: a retrospective cohort study. Reprod Biol Endocrinol. 2017;15: 67. doi:10.1186/s12958-017-0280-3

30. Morisaki N, Nagata C, Jwa SC, Sago H, Saito S, Oken E, et al. Pre-pregnancy BMI-specific optimal gestational weight gain for women in Japan. J Epidemiol. 2017;27: 492–498. doi:10.1016/j.je.2016.09.013

31. Ee TX, Allen Jr JC, Malhotra R, Koh H, Østbye T, Tan TC. Determining optimal gestational weight gain in a multiethnic Asian population. Journal of Obstetrics and Gynaecology Research. 2014;40: 1002–1008. doi:10.1111/jog.12307

32. Wang Y, Beydoun MA, Min J, Xue H, Kaminsky LA, Cheskin LJ. Has the prevalence of overweight, obesity and central obesity levelled off in the United States? Trends, patterns, disparities, and future projections for the obesity epidemic. International Journal of Epidemiology. 2020;49: 810–823. doi:10.1093/ije/dyz273

33. Wang MC, Freaney PM, Perak AM, Greenland P, Lloyd-Jones DM, Grobman WA, et al. Trends in Prepregnancy Obesity and Association With Adverse Pregnancy Outcomes in the United States, 2013 to 2018. Journal of the American Heart Association: Cardiovascular and Cerebrovascular Disease. 2021;10: e020717. doi:10.1161/JAHA.120.020717

34. Liang H, Yin C, Dong X, Acharya G, Li X. Clusters of week-specific maternal gestational weight gain pattern and their association with birthweight: an observational cohort study. Acta Obstetricia et Gynecologica Scandinavica. 2017;96: 1251–1260. doi:10.1111/aogs.13204

03 May 2025

Vidhura S Tennekoon
Academic Editor
PLOS ONE

Dear Dr Tennekoon

Re: Response to reviewer comments PLOS ONE-D-24-59010
Optimal gestational weight gain and pregnancy outcomes, by BMI and height, in a marginalised population of women with short stature living along the Thailand-Myanmar border: a retrospective cohort, 2004-2023.

On behalf of the authors of the above manuscript, I would like to submit a revised version of this manuscript. We thank the reviewers for their constructive comments which we feel has strengthened our manuscript. We have addressed all comments as specified in the bullet points overleaf. We have uploaded 2 new versions of the manuscript: one with tracked changes and one unmarked, and labelled them as prescribed by PLOS ONE.

The requested amended funding statement is as follows:
The Shoklo Malaria Research Unit is supported by the Wellcome-Trust Major Overseas Programme in Southeast Asia (#220211, lead applicant Nicholas Day) and in the years prior to that (WT-106698). MG was supported by a Rhodes Scholarship. For the purposes of Open Access, the author has applied a CC BY public copyright license to any Author Accepted Manuscript version arising from this submission. The funders had no role in study design, data collection and analysis, decision to publish, or preparation of the manuscript. There was no additional external funding received for this study.

We hope very much that this revised and improved manuscript will be acceptable for publication.

Yours sincerely,

Mary Gouws
DPhil Candidate, University of Oxford.
MSc International Health and Tropical Medicine, University of Oxford.
MBChB, University of Cape Town.

EDITORS' SPECIFIC POINTS:
Please find responses to comments in blue.

1. Please ensure that your manuscript meets PLOS ONE's style requirements, including those for file naming. The PLOS ONE style templates can be found at

The manuscript has been updated to meet PLOS ONE's style requirements and figures and supporting information have been saved with appropriate names and are now uploaded separately as individual files.

2. Thank you for stating in your Funding Statement: "The Shoklo Malaria Research Unit is supported in part by the Wellcome-Trust Major Overseas Programme in Southeast Asia (# 220211, https://doi.org/10.35802/220211; lead applicant Nicholas Day). For the purpose of Open Access, the author has applied a CC BY public copyright licence to any Author Accepted Manuscript version arising from this submission. There was no additional external funding received for this study. The funders had no role in study design, data collection and analysis, decision to publish, or preparation of the manuscript."
   Please provide an amended statement that declares *all* the funding or sources of support (whether external or internal to your organization) received during this study, as detailed online in our guide for authors at http://journals.plos.org/plosone/s/submit-now. Please also include the statement "There was no additional external funding received for this study." in your updated Funding Statement.
   Please include your amended Funding Statement within your cover letter. We will change the online submission form on your behalf.

We have amended the funding statement (below) and have included this revised version in the cover letter.
"The Shoklo Malaria Research Unit is supported by the Wellcome-Trust Major Overseas Programme in Southeast Asia (#220211, lead applicant Nicholas Day) and in the years prior to that (WT-106698). MG was supported by a Rhodes Scholarship. For the purposes of Open Access, the author has applied a CC BY public copyright license to any Author Accepted Manuscript version arising from this submission. The funders had no role in study design, data collection and analysis, decision to publish, or preparation of the manuscript. There was no additional external funding received for this study."

3. We note that you have indicated that there are restrictions to data sharing for this study. For studies involving human research participant data or other sensitive data, we encourage authors to share de-identified or anonymized data. However, when data cannot be publicly shared for ethical reasons, we allow authors to make their data sets available upon request. For information on unacceptable data access restrictions, please see http://journals.plos.org/plosone/s/data-availability#loc-unacceptable-data-access-restrictions.

Before we proceed with your manuscript, please address the following prompts:

a) If there are ethical or legal restrictions on sharing a de-identified data set, please explain them in detail (e.g., data contain potentially identifying or sensitive patient information, data are owned by a third-party organization, etc.) and who has imposed them (e.g., a Research Ethics Committee or Institutional Review

Board, etc.). Please also provide contact information for a data access committee, ethics committee, or other institutional body to which data requests may be sent.

b) If there are no restrictions, please upload the minimal anonymized data set necessary to replicate your study findings to a stable, public repository and provide us with the relevant URLs, DOIs, or accession numbers. Please see http://www.bmj.com/content/340/bmj.c181.long for guidelines on how to de-identify and prepare clinical data for publication. For a list of recommended repositories, please see https://journals.plos.org/plosone/s/recommended-repositories. You also have the option of uploading the data as Supporting Information files, but we would recommend depositing data directly to a data repository if possible.

Please update your Data Availability statement in the submission form accordingly.

The data availability statement now reads: "The data cannot be shared publicly due to ethical restrictions: this data was routinely collected from a marginalized population of undocumented refugees and migrants and the women have not consented for the data to be shared. These restrictions are in keeping with the policy of the Oxford Tropical Research Ethics Committee. However, de-identified data is available from the Mahidol-Oxford Research Unit institutional data access committee upon reasonable request from researchers who meet the criteria for access to confidential data (contact Rita Chanviriyavuth, email rita@tropmedres.ac)."

4. For studies involving third-party data, we encourage authors to share any data specific to their analyses that they can legally distribute. PLOS recognizes, however, that authors may be using third-party data they do not have the rights to share. When third-party data cannot be publicly shared, authors must provide all information necessary for interested researchers to apply to gain access to the data. (https://journals.plos.org/plosone/s/data-availability#loc-acceptable-data-access-restrictions)
   For any third-party data that the authors cannot legally distribute, they should include the following information in their Data Availability Statement upon submission:
   1) A description of the data set and the third-party source
   2) If applicable, verification of permission to use the data set
   3) Confirmation of whether the authors received any special privileges in accessing the data that other researchers would not have
   4) All necessary contact information others would need to apply to gain access to the data

This is not applicable for our study: third-party data was not involved in this analysis.

5. PLOS requires an ORCID iD for the corresponding author in Editorial Manager on papers submitted after December 6th, 2016. Please ensure that you have an ORCID iD and that it is validated in Editorial Manager. To do this, go to 'Update my Information' (in the upper left-hand corner of the main menu), and click on the Fetch/Validate link next to the ORCID field. This will take you to the ORCID site

and allow you to create a new iD or authenticate a pre-existing iD in Editorial Manager.

Rose McGready is the corresponding author, with ORCID iD: 0000-0003-1621-3257.

6. Please amend the manuscript submission data (via Edit Submission) to include author “Jacher Viladpai-Nguen”.

This has been updated accordingly, as requested.

RESPONSES TO REVIEWER COMMENTS

Reviewer #1: Thank for the paper, give us a good presentation, have an excellent proposed to help a vulnerable group of pregnant women. Nevertheless, I have a few suggestions before their publication.

Unify the objective (line 30-31) from the abstract with the last section of introduction (lines 71-72)

Amended as suggested.

Line 140. Is SES socioeconomic status? Clarify

SES is socioeconomic status and this is now spelled out in the methods.

Add the information to literacy and socioeconomic status as limitation (in Discussion section), because these variables can have influence on the health and well-being of the pregnant women and affect their GWG.

Amended as suggested (lines 285-288).

What is the reason to have greater intervale in those women with overweight-and obesity pregestational BMI (Table 3)? Add the information in discussion section.

This wider interval can be attributed to the wide variation of first trimester BMI in this overweight and obese group, ranging from 23 to 43kg/m² (a total range of 20 kg/m²). In the underweight BMI group, the range was only 4.9 kg/m² (13.5 to 18.4 kg/m²) and the range for the normal BMI category was bound at 4.4 kg/m² (18.5-22.9 kg/m²). We have added a few lines to discuss this, as suggested (lines 277-280) of the manuscript.

My final doubt is the applicability can be effective in all women with short stature? Regardless of the access to health service, and another sociocultural context? Or only those from Nepal, East Timor and Bangladesh?

There are limited data globally on women with short stature and as far as we are aware, no guidelines for GWG currently exist for these groups. Thus, the findings from our study currently provide the only insight for any pregnant woman with short stature, regardless of access to health services and sociocultural contexts. This has been clarified in the manuscript (lines 73-74 and 328-329).

Reviewer #2: Dear Authors,

Thank you for the opportunity to review this interesting piece of work, that highlights an under-adressed data gap in a marginalized population.

However, I have a few minor concerns and queries about the article:

1) Firstly, I suggest adjustments to the wordings in the article, particularly on the usage of "short stature women", as it may come across as stigmatizing this population, taking into consideration this population may have shorter stature due to a underlying varying genetic and/or environmental reasons. I suggest adhering to person-first

language i.e. "women with shorter stature" or "women with short stature" for a more neutral tone.
Thank you for this suggestion. Have amended the manuscript throughout to adhere to first person language.

2) I am curious as to why neonates with major congenital abnormalities were excluded from the analysis, considering maternal obesity is a known risk factor for congenital anomalies. If there is a strong reason to this, I suggest the authors include the explanation in the main text.
Thank you for raising this. The primary reason for this exclusion is that neonates with major congenital abnormalities often present with skewed birth weight distributions, such as being small or large for gestational age, which could confound the assessment of the relationship between maternal weight gain and neonatal outcomes. Additionally, certain congenital abnormalities can directly or indirectly influence gestational weight gain (GWG), further complicating the interpretation of results if included. As there was a relatively small number of neonates with congenital abnormalities, we anticipate that excluding this group should not substantially affect the results in any case. As suggested, ~~W~~we have added an explanation of this to the methods section (lines 109-112).

3) The women in the overweight/obese BMI range were analyzed as a homogenous group rather than 2 separate groups. The authors have alluded to the inherent issue of analyzing BMI as a categorical factor rather than continuous - that it oversimplifies risk estimation for women within a broad range of profiles such as BMI. In fact, the paper by Morisaki, quoted by the authors, found rather differing optimal GWG for women in the overweight (7.7kg) vs obese (4.3 kg) category. Therefore, I suggest the authors consider splitting the analysis of overweight (BMI 23-24.9 kg/m2) and obese (>25 kg/m2) categories, for better clinical applicability.
This is an important issue and we thank the reviewer for raising it. Although growing, the overweight and obese groups are still relatively small populations in this area. The small numbers meant that when separated, the samples were underpowered and we were not able to produce meaningful results. To demonstrate this more clearly, we have added Table 1 and 2 repeated but separated into overweight and obese groups in the supplementary materials (Table S1 and Table S2). The number of overweight BMI women was 2085 and 1975 for obese women. Additionally, the Asia-pacific classification of overweight BMI (BMI 23-24.9 kg/m2) is very narrow. We have mentioned this as a limitation in the discussion section.

4) I suggest p-values to be included in Tables 1 and 2, to reflect the baseline differences among different BMI categories, if any, as they may hold weight in interpreting the main outcome of the analysis.
Thank you for this suggestion. After consulting with the study statistician and in accordance with the STROBE guidelines for observational studies *(section 14, 2007 Explanation and Elaboration by* Vandenbroucke et al.) we note that descriptive tables are recommended to summarize participant characteristics without inferential statistics such as p-values. The rationale is to prevent any implication that these tables test hypotheses or draw conclusions about the data beyond simple description.

5) The authors addressed a potential overestimation of risk of adverse outcomes using the composite score - my question is how should the reader account for this when

applying the findings of the analysis in clinical practice. Should the clinician advise for a more lenient range, or perhaps upper-half of the normal range reported in this study, while advising optimal GWG for women with shorter stature? I suggest the authors to discuss the clinical implications of this.

We would suggest that a clinician in this setting should advise the same GWG range for all women within each BMI group, based on height. Thank you for this suggestion. To assess the potential overestimation of risk due to the composite outcome score, we examined the distribution of adverse outcomes. Because two thirds of our pregnancies 11,309 women (65.8%) had no adverse outcomes (n=11,309, 65.8%), these would have had the largest influence on the GWG estimations. Less than 4,878 (28.4%) had one, and 6% had more than 1 of the adverse outcomes indicating not only that it was uncommon for women to experience more than 1 adverse outcome, but also that those who did would have minimal impact on the estimated GWG recommendation. The frequencies of pregnancies experiencing no adverse outcome, 1 adverse outcome and 1 or more adverse outcomes is also now included in the results and more explanation has been included in the discussion as well1,007 (5.6%) experienced two or more. The number of women with 1 or more adverse outcomes has been added to the results (lines 215-217). However, although we were unable to run models for a full sensitivity analysis due to small sample sizes across BMI groups, we know that two-thirds of the women in this dataset had no adverse events therefore, the GWG recommendations favour this group. .

Reviewer #3: The study explored optimal gestational weight gain (GWG) by BMI and height in a marginalized migrant population along the Thailand-Myanmar border, aiming to inform more population-specific guidelines. Here are some of my concerns that require addressing:

The study does not distinguish between iatrogenic (medically indicated) and spontaneous preterm birth. Iatrogenic PTB (e.g., for preeclampsia or fetal distress) is often an appropriate and protective medical intervention rather than an adverse outcome. If iatrogenic PTB is grouped with spontaneous PTB, it could misrepresent GWG's true impact on PTB risk or the composite risk outcome.

Thank you for raising this point. In order to avoid the issues you mention, we have removed iatrogenic PTBs from the dataset (N=174) and rerun the analyses with the refined dataset (N=17194).

The study states that they adjusted for GDM screening method changes over time, but does not clearly define how GDM itself was accounted for in their models. A sensitivity analysis excluding women with GDM could strengthen the findings. This is hared across the other pregnancy complications including Malaria, and hypertensive disorder.

A sensitivity analysis excluding women with GDM was run and the GWG ranges were 10.2 to 14.8kg (compared with 10.2-14.8kg) for underweight women, 8.3 to 14.3kg (compared with 8.3 to 14.0kg) for normal weight women and 5.6 to 12.8kg (compared with 5.6 to 12.1kg) for overweight and obese women, i.e., there were no substantial changes to the results as currently presented.

In addition, we have now adjusted for year, and how GDM screening method changed over time in all models (explained in Statistical analysis section in Methods).

Parity status - intriguing the majority of women were obese were multiparous. Nulliparity is what essentially creates a "unknown" risk - if you had "normal

deliveries/pregnancies" before then that is the best predictor about cows in future pregnancies. I would be interested in a nulliparous analysis being highlighted. Similarly, inter pregnancy weight gain - if that is something that can be teased out and its associated risk with the composite risk - that is very interesting.

We agree that both these points would be interesting topics to pursue. We have run the analysis in nulliparous women (N=6231) only, and while slightly lower for the underweight and normal BMI groups, the results were not substantially different from the results of the total cohort:

- Underweight BMI group: 10.0-14.3kg (N=1345)
- Normal BMI group: 7.7-12.7kg (N=3933)
- Overweight/obese BMI group: 4.9-11.3 (N=953)

As the differences were minimal, and guideline implementation is already challenging in particular in resource-limited settings, at present we believe that it would not be practical to implement different recommendations across multiple groups. If recommendations become too complicated, the trade-off is often that they are simply not implemented. The next plan is to invite the frontline staff in this resource-limited setting to participate to a working group to determine their perceptions of implementing GWG guidelines, including whether it would be clinically feasible to implement appropriate separate recommendations by parity status.

Unfortunately, we do not have data on interpregnancy weight gain but will keep it mind for future research projects!

I assume - although. this is not spelt out by the authors - that some women here would feature multiple times? If you only included one pregnancy which one? Or how were the repeat pregnancies handled—were they accounted for in the statistical models?

Ideally, we would ~~have~~ like to adjust~~ed~~ for repeat~~ed~~ pregnancies (non-independence of data)~~ within individuals~~. However, data on repeat pregnancies only started being collected from 2011/2012 (with site by site initiation) which meant that ~~so many data are missing for the earlier years (~~more than 9,000 pregnancies did not have this information~~women)~~.

~~Among women for whom repeat pregnancy information was available, 743 pregnancies were repeat pregnancies.However, if we were to adjust for repeat pregnancies, it would not be clear if the differences would be attributable to repeat pregnancies or due to changes over time. We therefore did not adjust for repeat pregnancies, but w~~We have added several sentences to the discussion mentioning this as a ~~potential~~ limitation (lines 289-291). ~~.~~

Commented [SL1]: Out of how many?

The authors use the Perinatal Institute’s Gestation Related Optimal Weight (GROW) customised bulk centile calculator V.8.0[18]. They state: GROW offers the advantage of region-specific classification and has coefficients to represent over 100 country-of-origin groups. -> which ethnic group did you choose? and do justify the choice.

Apologies that this was not clearly stated. For women with Burmese ethnicity, the Myanmar standards were used. For Karen and other ethnicities, Nepalese standards were used as the average height of Karen and other ethnicities in the dataset most closely matched the average height of Nepalese women. The methods section has been revised to include this information.

Strengths:
Addresses a population often underrepresented in research.
Attempts to tailor GWG recommendations to short-stature women, which is clinically relevant.
Uses GROW centiles, which allow for ethnicity-specific fetal growth assessment.
Utilizes modern statistical methods (natural cubic splines) to model nonlinear relationships.
Thank you!

Key Limitations to Address Before Publication:
Clarify whether multiple pregnancies from the same individuals were included and account for within-subject correlation.
Addressed, please see above.

Differentiate between iatrogenic vs. spontaneous PTB.
Addressed, please see above.

Explicitly describe how GDM was adjusted for in the analysis.
Addressed, please see above.

Provide further justification for cubic spline modelling choices and potential overfitting concerns.
We have adjusted the text in the methods to read: "As visual inspection of the association between GWG and adverse outcomes indicated nonlinear relationships, natural cubic splines with logistic regression models were used. Natural cubic splines were used to mitigate overfitting, especially at the boundaries of the data." And the following reference that offers support for this approach is cited: Gauthier J, Wu QV, Gooley TA. Cubic splines to model relationships between continuous variables and outcomes: a guide for clinicians. Bone Marrow Transplant. 2020;55: 675–680. doi:10.1038/s41409-019-0679-x~~As visual inspection of the association between GWG and adverse outcomes indicated nonlinear relationships, natural cubic splines were used.~~

Commented [SL2]: Hi mary, there is also a reference cited here that looks like it is about multilevel modelling which I don't think we used for this analysis so I'm not sure this reference is relevant?
(Snijders TAB, Bosker RJ. Multilevel Analysis: An Introduction to Basic and Advanced Multilevel Modeling. SAGE; 2011.)
