# Supplementary material for: Optimal gestational weight gain and pregnancy outcomes, by BMI and height, in a marginalised population of women with short stature living along the Thailand-Myanmar border: A retrospective cohort, 2004–2023
Source: PLoS One. 2025 Oct 3;20(10):e0330256. doi: 10.1371/journal.pone.0330256 (PMC12494276; doi:10.1371/journal.pone.0330256)
Supplement: Attachment — Submitted filename: Reviewer_rebuttal_letter_May2025_FINAL.docx [file pone.0330256.s004.docx]

03 May 2025

Vidhura S Tennekoon
Academic Editor

PLOS ONE

Dear Dr Tennekoon

**Re: Response to reviewer comments PLOS ONE-D-24-59010**

Optimal gestational weight gain and pregnancy outcomes, by BMI and height, in a marginalised population of women with short stature living along the Thailand-Myanmar border: a retrospective cohort, 2004-2023.

On behalf of the authors of the above manuscript, I would like to submit a revised version of this manuscript. We thank the reviewers for their constructive comments which we feel has strengthened our manuscript. We have addressed all comments as specified in the bullet points overleaf. We have uploaded 2 new versions of the manuscript: one with tracked changes and one unmarked, and labelled them as prescribed by PLOS ONE.

The requested amended funding statement is as follows:

The Shoklo Malaria Research Unit is supported by the Wellcome-Trust Major Overseas Programme in Southeast Asia (#220211, lead applicant Nicholas Day) and in the years prior to that (WT-106698). MG was supported by a Rhodes Scholarship. For the purposes of Open Access, the author has applied a CC BY public copyright license to any Author Accepted Manuscript version arising from this submission. The funders had no role in study design, data collection and analysis, decision to publish, or preparation of the manuscript. There was no additional external funding received for this study.

We hope very much that this revised and improved manuscript will be acceptable for publication.

Yours sincerely,

Mary Gouws

DPhil Candidate, University of Oxford.

MSc International Health and Tropical Medicine, University of Oxford.

MBChB, University of Cape Town.

**EDITORS' SPECIFIC POINTS:**

*Please find responses to comments in blue.*

1. Please ensure that your manuscript meets PLOS ONE's style requirements, including those for file naming. The PLOS ONE style templates can be found at

The manuscript has been updated to meet PLOS ONE’s style requirements and figures and supporting information have been saved with appropriate names and are now uploaded separately as individual files.

2. Thank you for stating in your Funding Statement: “The Shoklo Malaria Research Unit is supported in part by the Wellcome-Trust Major Overseas Programme in Southeast Asia (# 220211, <https://doi.org/10.35802/220211;> lead applicant Nicholas Day). For the purpose of Open Access, the author has applied a CC BY public copyright licence to any Author Accepted Manuscript version arising from this submission. There was no additional external funding received for this study. The funders had no role in study design, data collection and analysis, decision to publish, or preparation of the manuscript.”

Please provide an amended statement that declares *all* the funding or sources of support (whether external or internal to your organization) received during this study, as detailed online in our guide for authors at <http://journals.plos.org/plosone/s/submit-now>.  Please also include the statement “There was no additional external funding received for this study.” in your updated Funding Statement.

Please include your amended Funding Statement within your cover letter. We will change the online submission form on your behalf.

We have amended the funding statement (below) and have included this revised version in the cover letter.

“The Shoklo Malaria Research Unit is supported by the Wellcome-Trust Major Overseas Programme in Southeast Asia (#220211, lead applicant Nicholas Day) and in the years prior to that (WT-106698). MG was supported by a Rhodes Scholarship. For the purposes of Open Access, the author has applied a CC BY public copyright license to any Author Accepted Manuscript version arising from this submission. The funders had no role in study design, data collection and analysis, decision to publish, or preparation of the manuscript. There was no additional external funding received for this study.”

3. We note that you have indicated that there are restrictions to data sharing for this study. For studies involving human research participant data or other sensitive data, we encourage authors to share de-identified or anonymized data. However, when data cannot be publicly shared for ethical reasons, we allow authors to make their data sets available upon request. For information on unacceptable data access restrictions, please see <http://journals.plos.org/plosone/s/data-availability#loc-unacceptable-data-access-restrictions>.

Before we proceed with your manuscript, please address the following prompts:

a) If there are ethical or legal restrictions on sharing a de-identified data set, please explain them in detail (e.g., data contain potentially identifying or sensitive patient information, data are owned by a third-party organization, etc.) and who has imposed them (e.g., a Research Ethics Committee or Institutional Review Board, etc.). Please also provide contact information for a data access committee, ethics committee, or other institutional body to which data requests may be sent.
b) If there are no restrictions, please upload the minimal anonymized data set necessary to replicate your study findings to a stable, public repository and provide us with the relevant URLs, DOIs, or accession numbers. Please see <http://www.bmj.com/content/340/bmj.c181.long> for guidelines on how to de-identify and prepare clinical data for publication. For a list of recommended repositories, please see <https://journals.plos.org/plosone/s/recommended-repositories>. You also have the option of uploading the data as Supporting Information files, but we would recommend depositing data directly to a data repository if possible.

Please update your Data Availability statement in the submission form accordingly.

The data availability statement now reads: “The data cannot be shared publicly due to ethical restrictions: this data was routinely collected from a marginalized population of undocumented refugees and migrants and the women have not consented for the data to be shared. These restrictions are in keeping with the policy of the Oxford Tropical Research Ethics Committee. However, de-identified data is available from the Mahidol-Oxford Research Unit institutional data access committee upon reasonable request from researchers who meet the criteria for access to confidential data (contact Rita Chanviriyavuth, email [rita@tropmedres.ac](mailto:rita@tropmedres.ac)).”

4. For studies involving third-party data, we encourage authors to share any data specific to their analyses that they can legally distribute. PLOS recognizes, however, that authors may be using third-party data they do not have the rights to share. When third-party data cannot be publicly shared, authors must provide all information necessary for interested researchers to apply to gain access to the data. (<https://journals.plos.org/plosone/s/data-availability#loc-acceptable-data-access-restrictions>)

For any third-party data that the authors cannot legally distribute, they should include the following information in their Data Availability Statement upon submission:
1) A description of the data set and the third-party source
2) If applicable, verification of permission to use the data set
3) Confirmation of whether the authors received any special privileges in accessing the data that other researchers would not have
4) All necessary contact information others would need to apply to gain access to the data

This is not applicable for our study: third-party data was not involved in this analysis.

5. PLOS requires an ORCID iD for the corresponding author in Editorial Manager on papers submitted after December 6th, 2016. Please ensure that you have an ORCID iD and that it is validated in Editorial Manager. To do this, go to ‘Update my Information’ (in the upper left-hand corner of the main menu), and click on the Fetch/Validate link next to the ORCID field. This will take you to the ORCID site and allow you to create a new iD or authenticate a pre-existing iD in Editorial Manager.

Rose McGready is the corresponding author, with ORCID iD: 0000-0003-1621-3257.

6. Please amend the manuscript submission data (via Edit Submission) to include author “Jacher Viladpai-Nguen”.

This has been updated accordingly, as requested.

**RESPONSES TO REVIEWER COMMENTS**

**Reviewer #1: Thank for the paper, give us a good presentation, have an excellent proposed to help a vulnerable group of pregnant women. Nevertheless, I have a few suggestions before their publication.**

Unify the objective (line 30-31) from the abstract with the last section of introduction (lines 71-72)

Amended as suggested.

Line 140. Is SES socioeconomic status? Clarify

SES is socioeconomic status and this is now spelled out in the methods.

Add the information to literacy and socioeconomic status as limitation (in Discussion section), because these variables can have influence on the health and well-being of the pregnant women and affect their GWG.

Amended as suggested (lines 285-288).

What is the reason to have greater intervale in those women with overweight-and obesity pregestational BMI (Table 3)? Add the information in discussion section.

This wider interval can be attributed to the wide variation of first trimester BMI in this overweight and obese group, ranging from 23 to 43kg/m² (a total range of 20 kg/m²). In the underweight BMI group, the range was only 4.9 kg/m² (13.5 to 18.4 kg/m²) and the range for the normal BMI category was bound at 4.4 kg/m² (18.5-22.9 kg/m²). We have added a few lines to discuss this, as suggested (lines 277-280) of the manuscript.

My final doubt is the applicability can be effective in all women with short stature? Regardless of the access to health service, and another sociocultural context? Or only those from Nepal, East Timor and Bangladesh?

There are limited data globally on women with short stature and as far as we are aware, no guidelines for GWG currently exist for these groups. Thus, the findings from our study currently provide the only insight for any pregnant woman with short stature, regardless of access to health services and sociocultural contexts. This has been clarified in the manuscript (lines 73-74 and 328-329).

**Reviewer #2: Dear Authors,
Thank you for the opportunity to review this interesting piece of work, that highlights an under-adressed data gap in a marginalized population.**

However, I have a few minor concerns and queries about the article:

1) Firstly, I suggest adjustments to the wordings in the article, particularly on the usage of "short stature women", as it may come across as stigmatizing this population, taking into consideration this population may have shorter stature due to a underlying varying genetic and/or environmental reasons. I suggest adhering to person-first language i.e. "women with shorter stature" or "women with short stature" for a more neutral tone.

Thank you for this suggestion. Have amended the manuscript throughout to adhere to first person language.

2) I am curious as to why neonates with major congenital abnormalities were excluded from the analysis, considering maternal obesity is a known risk factor for congenital anomalies. If there is a strong reason to this, I suggest the authors include the explanation in the main text.

Thank you for raising this. The primary reason for this exclusion is that neonates with major congenital abnormalities often present with skewed birth weight distributions, such as being small or large for gestational age, which could confound the assessment of the relationship between maternal weight gain and neonatal outcomes. Additionally, certain congenital abnormalities can directly or indirectly influence gestational weight gain (GWG), further complicating the interpretation of results if included. As there was a relatively small number of neonates with congenital abnormalities, we anticipate that excluding this group should not substantially affect the results in any case. As suggested, we have added an explanation of this to the methods section (lines 109-112).

3) The women in the overweight/obese BMI range were analyzed as a homogenous group rather than 2 separate groups. The authors have alluded to the inherent issue of analyzing BMI as a categorical factor rather than continuous - that it oversimplifies risk estimation for women within a broad range of profiles such as BMI. In fact, the paper by Morisaki, quoted by the authors, found rather differing optimal GWG for women in the overweight (7.7kg) vs obese (4.3 kg) category. Therefore, I suggest the authors consider splitting the analysis of overweight (BMI 23-24.9 kg/m2) and obese (>25 kg/m2) categories, for better clinical applicability.

This is an important issue and we thank the reviewer for raising it. Although growing, the overweight and obese groups are still relatively small populations in this area. The small numbers meant that when separated, the samples were underpowered and we were not able to produce meaningful results. To demonstrate this more clearly, we have added Table 1 and 2 repeated but separated into overweight and obese groups in the supplementary materials (Table S1 and Table S2). The number of overweight BMI women was 2085 and 1975 for obese women. Additionally, the Asia-pacific classification of overweight BMI (BMI 23-24.9 kg/m2) is very narrow. We have mentioned this as a limitation in the discussion section.

4) I suggest p-values to be included in Tables 1 and 2, to reflect the baseline differences among different BMI categories, if any, as they may hold weight in interpreting the main outcome of the analysis.

Thank you for this suggestion. After consulting with the study statistician and in accordance with the STROBE guidelines for observational studies *(section 14, 2007 Explanation and Elaboration by* Vandenbroucke et al.) we note that descriptive tables are recommended to summarize participant characteristics without inferential statistics such as p-values. The rationale is to prevent any implication that these tables test hypotheses or draw conclusions about the data beyond simple description.

5) The authors addressed a potential overestimation of risk of adverse outcomes using the composite score - my question is how should the reader account for this when applying the findings of the analysis in clinical practice. Should the clinician advise for a more lenient range, or perhaps upper-half of the normal range reported in this study, while advising optimal GWG for women with shorter stature? I suggest the authors to discuss the clinical implications of this.

We would suggest that a clinician in this setting should advise the same GWG range for all women within each BMI group, based on height. Because two thirds of our pregnancies had no adverse outcomes (n=11,309, 65.8%), these would have had the largest influence on the GWG estimations. Less than 6% had more than 1 of the adverse outcomes indicating not only that it was uncommon for women to experience more than 1 adverse outcome, but also that those who did would have minimal impact on the estimated GWG recommendation. The frequencies of pregnancies experiencing no adverse outcome, 1 adverse outcome and 1 or more adverse outcomes is also now included in the results and more explanation has been included in the discussion as well.

**Reviewer #3: The study explored optimal gestational weight gain (GWG) by BMI and height in a marginalized migrant population along the Thailand-Myanmar border, aiming to inform more population-specific guidelines. Here are some of my concerns that require addressing:**

The study does not distinguish between iatrogenic (medically indicated) and spontaneous preterm birth. Iatrogenic PTB (e.g., for preeclampsia or fetal distress) is often an appropriate and protective medical intervention rather than an adverse outcome. If iatrogenic PTB is grouped with spontaneous PTB, it could misrepresent GWG’s true impact on PTB risk or the composite risk outcome.

Thank you for raising this point. In order to avoid the issues you mention, we have removed iatrogenic PTBs from the dataset (N=174) and rerun the analyses (N=17194).

The study states that they adjusted for GDM screening method changes over time, but does not clearly define how GDM itself was accounted for in their models. A sensitivity analysis excluding women with GDM could strengthen the findings. This is hared across the other pregnancy complications including Malaria, and hypertensive disorder.

A sensitivity analysis excluding women with GDM was run and the GWG ranges were 10.2 to 14.8kg (compared with 10.2-14.8kg) for underweight women, 8.3 to 14.3kg (compared with 8.3 to 14.0kg) for normal weight women and 5.6 to 12.8kg (compared with 5.6 to 12.1kg) for overweight and obese women, i.e., there were no substantial changes to the results as currently presented.

In addition, we have now adjusted for year, and how GDM screening method changed over time in all models (explained in Statistical analysis section in Methods).

Parity status - intriguing the majority of women were obese were multiparous. Nulliparity is what essentially creates a "unknown" risk - if you had "normal deliveries/pregnancies" before then that is the best predictor about cows in future pregnancies. I would be interested in a nulliparous analysis being highlighted. Similarly, inter pregnancy weight gain - if that is something that can be teased out and its associated risk with the composite risk - that is very interesting.

We agree that both these points would be interesting topics to pursue. We have run the analysis in nulliparous women (N=6231) only, and while slightly lower for the underweight and normal BMI groups, the results were not substantially different from the results of the total cohort:

- Underweight BMI group: 10.0-14.3kg (N=1345)
- Normal BMI group: 7.7-12.7kg (N=3933)
- Overweight/obese BMI group: 4.9-11.3 (N=953)

As the differences were minimal, and guideline implementation is already challenging in particular in resource-limited settings, at present we believe that it would not be practical to implement different recommendations across multiple groups. If recommendations become too complicated, the trade-off is often that they are simply not implemented. The next plan is to invite the frontline staff in this resource-limited setting to participate to a working group to determine their perceptions of implementing GWG guidelines, including whether it would be clinically feasible to implement appropriate separate recommendations by parity status.

Unfortunately, we do not have data on interpregnancy weight gain but will keep it mind for future research projects!

I assume - although. this is not spelt out by the authors - that some women here would feature multiple times? If you only included one pregnancy which one? Or how were the repeat pregnancies handled—were they accounted for in the statistical models?

Ideally, we would like to adjust for repeat pregnancies (non-independence of data). However, data on repeat pregnancies only started being collected from 2011/2012 (with site by site initiation) which meant that more than 9,000 pregnancies did not have this information. We have added several sentences to the discussion mentioning this as a limitation (lines 289-291).

The authors use the Perinatal Institute’s Gestation Related Optimal Weight (GROW) customised bulk centile calculator V.8.0[18]. They state: GROW offers the advantage of region-specific classification and has coefficients to represent over 100 country-of-origin groups. -> which ethnic group did you choose? and do justify the choice.

Apologies that this was not clearly stated. For women with Burmese ethnicity, the Myanmar standards were used. For Karen and other ethnicities, Nepalese standards were used as the average height of Karen and other ethnicities in the dataset most closely matched the average height of Nepalese women. The methods section has been revised to include this information.

Strengths:
Addresses a population often underrepresented in research.
Attempts to tailor GWG recommendations to short-stature women, which is clinically relevant.
Uses GROW centiles, which allow for ethnicity-specific fetal growth assessment.
Utilizes modern statistical methods (natural cubic splines) to model nonlinear relationships.

Thank you!

Key Limitations to Address Before Publication:
Clarify whether multiple pregnancies from the same individuals were included and account for within-subject correlation.

Addressed, please see above.

Differentiate between iatrogenic vs. spontaneous PTB.

Addressed, please see above.

Explicitly describe how GDM was adjusted for in the analysis.

Addressed, please see above.

Provide further justification for cubic spline modelling choices and potential overfitting concerns.

We have adjusted the text in the methods to read: “As visual inspection of the association between GWG and adverse outcomes indicated nonlinear relationships, natural cubic splines with logistic regression models were used. Natural cubic splines were used to mitigate overfitting, especially at the boundaries of the data.” And the following reference that offers support for this approach is cited: Gauthier J, Wu QV, Gooley TA. Cubic splines to model relationships between continuous variables and outcomes: a guide for clinicians. Bone Marrow Transplant. 2020;55: 675–680. doi:10.1038/s41409-019-0679-x
