# Supplementary material for: Optimal gestational weight gain and pregnancy outcomes, by BMI and height, in a marginalised population of women with short stature living along the Thailand-Myanmar border: A retrospective cohort, 2004–2023
Source: PLoS One. 2025 Oct 3;20(10):e0330256. doi: 10.1371/journal.pone.0330256 (PMC12494276; doi:10.1371/journal.pone.0330256)
Supplement: Attachment — Submitted filename: Response to reviewers.docx [file pone.0330256.s005.docx]

25 July 2025

Vidhura S Tennekoon
Academic Editor

PLOS ONE

Dear Dr Tennekoon,

**Re: Response to reviewer comments PLOS ONE-D-24-59010_R1**

Optimal gestational weight gain and pregnancy outcomes, by BMI and height, in a marginalised population of women with short stature living along the Thailand-Myanmar border: a retrospective cohort, 2004-2023.

On behalf of the authors of the above manuscript, I would like to submit a revised version of this manuscript. We thank you for the opportunity to improve the manuscript before publication. All comments have been addressed as specified; please see responses below. Two new versions of the manuscript have been uploaded: one that highlights the changes made and one without tracked changes.

We hope very much that this revised and improved manuscript will be acceptable for publication.

Yours sincerely,

Prof Rose McGready

**RESPONSES TO COMMENTS** from **REVIEWER 2**

**1) As the main focus on this study is on women with short stature - there should be an explicit statement of why 153 cm was taken as the threshold in this study.**

The height of <153cm was chosen as the cut-off because ≥153cm was used by the INTERGROWTH-21 project as one of the factors “commonly used to identify women who would benefit from low-risk, routine antenatal care”:

- Cheikh Ismail L, Bishop DC, Pang R, et al. Gestational Weight Gain Standards Based on Women Enrolled in the Fetal Growth Longitudinal Study of the INTERGROWTH-21st Project: A Prospective Longitudinal Cohort Study. BMJ 2016, 352, i555, doi:10.1136/bmj.i555
- Villar J, Altman DG, Purwar M, et al. International Fetal and Newborn Growth Consortium for the 21st Century. The objectives, design and implementation of the INTERGROWTH-21st Project. BJOG 2013;120(suppl 2):9-26, v. doi:10.1111/1471-0528.12047

To clarify why this cut-off was chosen, we have added the following line in the methods: “Similar to the IG-21study, short stature was defined as maternal height less than 153cm.”

**2) There should be discussions on the differential background metabolic risks of populations with short stature, in relation to BMI, compared to taller populations. Underlying genetic, environmental, and socioeconomic factors that may confound short stature and adverse pregnancy outcomes should be highlighted as well.**

Thank you for this suggestion. We agree that the relationship between maternal height, BMI and adverse pregnancy outcomes is complicated. We have added a paragraph to the discussion to highlight the complexity that needs to be considered, The relationship between maternal height, birth outcomes and BMI is complex. The women in this study represent a marginalised population where nutrition may be compromised but there is also an increasing proportion of women with overweight or obese BMIs[35]. This “double burden of malnutrition”[36] combined with short stature may impact the outcomes considered in this study, including caesarean section[37,38], small for gestational age and large for gestational age[39]. Indeed, Rahman et al reported that maternal overweight combined with short stature was associated with a higher risk of caesarean section regardless of socio-economic status which usually predicts risk for this outcome[37]. Yearwood et al found maternal height modified the association between SGA and LGA and risk of adverse outcomes; increased maternal height was associated with increased adverse outcomes in SGA babies but decreased risk in LGA babies[39]. Although we know that all women in this cohort were seeking services in a humanitarian healthcare setting, data on literacy and socioeconomic status, which can influence health and well-being of pregnant women and affect the GWG, was limited for this cohort, thus could not be adjusted for in the analysis.”

**3) The authors should caution from stating that these findings can be generalized to other populations.**

We agree that readers should be cautious about the generalisability of our results. For this reason we have purposefully used cautious language such as “The findings from our study MAY also be applicable…”. However, we also believe it is important to note that there are limited data globally on women with short stature and as far as we are aware, no guidelines for GWG currently exist for these groups. Therefore, the findings from our study currently provide the only insight for any pregnant woman with short stature, regardless of access to health services and sociocultural contexts and we have presented our results in this context.

**4) There are other minor formatting issues highlighted in comments in the document.**

Apologies for the formatting errors. The extra table that was inserted between the Figure headings/legends has now been removed. The table title for Table 3 has been modified to “Calculated optimal GWG ranges based on minimisation of composite risk of adverse outcomes, per Asia-Pacific BMI category, by height (<153cm vs. ≥153cm) and compared with NAM GWG recommendations”. The n for the full cohort has been added to Table 3. All abbreviations are now defined for all tables. In the discussion, where there was repetition of the previous sentence, we have removed “where only a small proportion of women fall into the overweight/obese category”.

In addition, a sentence has been added to the discussion stating that another reason for the differences found may be due to the pregnancy outcomes that were considered in our study compared with those considered for the NAM GWG recommendations, “One reason for this difference may be because while this study considered the outcomes of HDoP, GDM, CS, spontaneous preterm birth, SGA and LGA, the NAM GWG recommendations additionally considered postpartum weight retention and childhood obesity, but not GDM [7]. Data on postpartum weight retention and childhood obesity were not available for analysis in this study.”
